# Glucocorticoid-induced Leucine Zipper (GILZ) is a novel secreted protein by intestinal L-cells and is dysregulated during active ulcerative colitis

**DOI:** 10.1038/s41420-026-03204-w

**Published:** 2026-06-17

**Authors:** Lucrezia Rosati, Giuseppe Leoncini, Luigi Cari, Maria Rosaria Sette, Martina Procaccini, Giuseppe Nocentini, Vincenzo Villanacci, Carlo Riccardi, Graziella Migliorati, Simona Ronchetti

**Affiliations:** 1 Pharmacology Division, Department of Medicine and Surgery, University of Perugia, Perugia, Italy; 2 First Pathology Division, Department of Services and Advanced Diagnostics, Fondazione IRCCS Istituto Nazionale dei Tumori, Milan, Italy; 3 Institute of Pathology, ASST Spedali Civili, Brescia, Italy

**Keywords:** Ulcerative colitis, Inflammation, Colon

## Abstract

The Glucocorticoid-Induced Leucine Zipper (GILZ) is a key mediator of the anti-inflammatory effects of glucocorticoids, primarily within the immune system. Recent evidence has implicated GILZ as a secretive protein in goblet cells, with reduced expression linked to active Inflammatory Bowel Disease (IBD), suggesting a role in intestinal cell homeostasis. In this context, GILZ has been found also in enteroendocrine cells (EEC), but its role remained undefined. This study aimed at identifying GILZ-expressing EEC subtypes, dissecting how the secretive function is affected by inflammation in human ulcerative colitis (UC) and exploring its role in these cells. GILZ was predominantly expressed in glucagon-like-peptide-1 (GLP-1)-secreting L-cells, across all stages of EEC differentiation. GILZ was also expressed in serotonin (5HT)-producing enterochromaffin cells (EC), even though at low levels. Such an expression profile was supported by analysis of publicly available single-cell RNA sequencing datasets, identifying both EEC progenitors and mature subsets. Interestingly, confocal immunofluorescence localized GILZ to cytoplasmic granules partially co-staining with GLP-1-containing vesicles. Histological analysis of mucosal colonic biopsies revealed a global reduction in EEC during active UC as compared to healthy individuals and quiescent UC. Specifically, GILZ-expressing L-cells were significantly reduced in active UC and only partially restored in quiescent disease. In contrast, 5HT-producing EC cells were still reduced during active UC but fully recovered in quiescent disease. In vitro, NCI-H716 L-cell line-secreted products reduced IL-8 secretion in Caco2 epithelial cells, indicating an anti-inflammatory effect. This activity was attenuated following GILZ silencing. Interestingly, treatment with a recombinant TAT-GILZ protein directly diminished IL-8 in Caco2 cells. Collectively, our findings identify GILZ as a novel secretory product of L-cells with potential anti-inflammatory properties. Restoring GILZ secretion may represent a promising therapeutic strategy to mitigate chronic intestinal inflammation in UC.

## Introduction

GILZ is a glucocorticoid-induced gene that mimics several anti-inflammatory effects of glucocorticoids in immune cells, as reported by studies in pre-clinical models of chronic/inflammatory diseases, including colitis [1–10]. The recent identification of GILZ as a secretive protein in human goblet cells has broaden the array of its functional implications, opening considerations on its secretive role into GI tract and beyond. While reduced expression of GILZ in goblet cells has been correlated with neutrophil mucosal infiltration and disease activity in IBD patients, the observation of GILZ expression in EEC remained unexplored [11].

EEC are epithelial cells interspersed throughout the GI mucosa, which are triggered to release neuroendocrine peptides and hormones into the *lamina propria* after stimulation by bacterial metabolites and food-derived molecules [12, 13]. In addition, EEC display neuronal axon-like properties, producing vesicles for synaptic transmission [14, 15]. Once delivered into the *lamina propria*, hormones can act on both local cells and neurons (*paracrine* mode), as well as on distant cellular targets, exploiting either the blood stream (*endocrine* mode) or the synaptic transmission (*synaptic* mode) [16]. Although the density of EEC is typically low throughout the GI mucosa, accounting for about 1% of the epithelial cells, they constitute the largest endocrine organ capable to link the gut to extra-intestinal organs, including the brain [17, 18]. Currently, the use of organoid-based platform of human EECs have contributed to bridge the gap in the study of EEC secretome, leading to the release of an atlas of human EEC subtypes [19]. New peptides that are secreted by EEC cells have been identified, but the list is still incomplete [20]. Furthermore, recent single cell studies revealed that human EEC secreted peptides not always overlapped with their murine counterpart [21].

Colonic EEC differentiate from pluripotent Lrg5^+^ stem cells, which are located deep into the crypt. Lineage differentiation proceeds via activation of Math1, Neurogenin 3 (NGN3), and Neurogenic differentiation factors [22, 23]. A recent classification subdivided colonic EEC into enterochromaffin (EC) cells, which are responsible for serotonin (5HT) secretion, and L-cells, identified as pro-glucagon and peptide YY (PYY) source [24]. Pro-glucagon is further processed into glucagon-like peptide 1 (GLP-1) and GLP-2. GLP-1 is involved in glucose metabolism, inhibition of gastric emptying, negative regulation of food intake and body weight, whereas GLP-2 promotes crypt cell proliferation, intestinal stem cell expansion, and intestinal growth [17, 25, 26].

IBD is a clinical term including ulcerative colitis (UC) and Crohn’s disease (CD), which are chronic relapsing diseases affecting the patients’ quality of life, also representing an economic burden worldwide. The propensity to relapse and disease-related complications are the major challenges to face in the clinical management and treatment planning of IBD patients [27]. Pharmacological treatments include glucocorticoids, non-steroidal anti-inflammatory drugs (NSAID), azathioprine and biologic agents. Nonetheless, achieving deep clinical remission still remains elusive [28, 29]. The impact of inflammatory processes on EEC is currently a matter of debate. While some Authors have been referring to IBD as a potential risk factor for neuroendocrine cell proliferations, ranging from neuroendocrine hyperplasia and micronests to neuroendocrine tumors (NET), others have seen no significant association between IBD and neuroendocrine proliferation [30–33]. On the other hand, the influence of EEC on immune homeostasis has been proposed and the loss of EEC in diseases leading to gut dysfunctions corroborated that hypothesis. Such a critical role of EEC is further supported by the finding of abnormal permeability and inflammatory signature in experimental studies with EEC-deficient human organoids, underscoring their function as key regulators of intestinal inflammation [24, 34–40].

The present study aimed at characterizing the expression and distribution of GILZ in distinct EEC subtypes, in both healthy individuals and UC patients. GILZ was found to be predominantly expressed in L-cells, including both precursors and mature cell populations. GILZ was also localized to intracellular vesicles partially co-expressing GLP-1 and Synaptophysin. These findings were confirmed by interrogating transcriptomic datasets. Immunofluorescence analysis revealed a reduction in the number of EEC, both L-cells and EC, in active UC. To investigate the functional role of GILZ in L-cells, in vitro experiments with the human L-cell line NCI-H716 and epithelial Caco2 cells demonstrated GILZ to be a new protein secreted by L-cells acting as a potential anti-inflammatory mediator.

## Results

### GILZ is expressed in distinct subtypes of EEC in the human colon

**Fig. 1:**
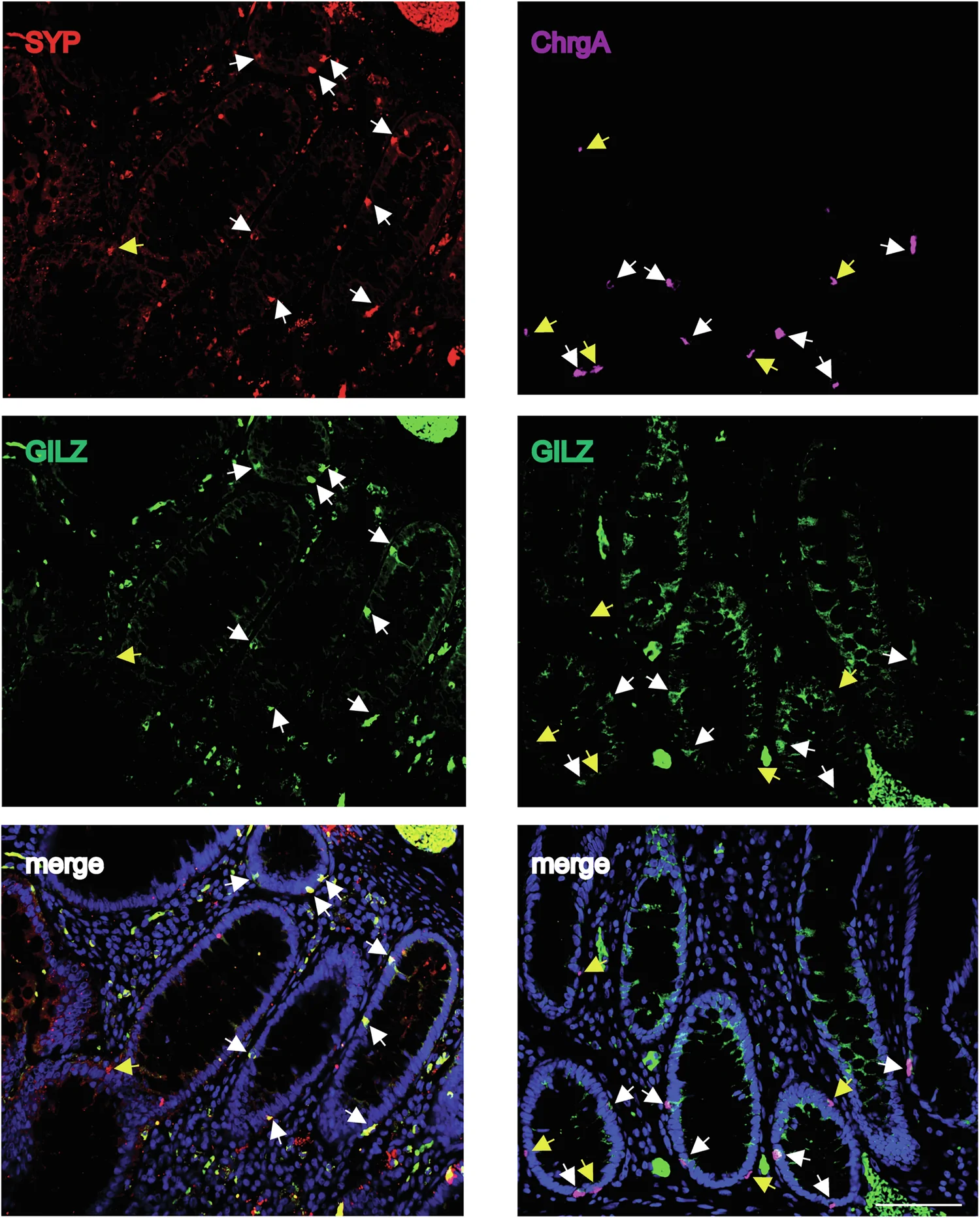
Representative confocal microscopy images of either anti-SYP plus anti-GILZ Ab (left panels) or anti-ChrgA plus anti-GILZ Ab (right panels) staining in human colon sections. EEC were identified with either SYP or ChrgA immunoreactivity (both white and yellow arrows). Yellow arrows indicate EEC not expressing GILZ (GILZ^-^). DAPI staining identifies nuclei. Scale bar: 100 µm.

GILZ expression has been previously established in human goblet cells by our group [11]. We also observed that GILZ was expressed in the cytoplasm of other cells, which exhibited apico-basal polarity by immunofluorescence staining (Supplementary Fig. 1). In a co-staining with GILZ, EEC were identified as immunoreactive both for synaptophysin (SYP) and Chromogranin A (ChgA). The results demonstrated the presence of GILZ in almost all EEC (Fig. 1, white arrowheads), even though isolated EEC resulted GILZ negative (GILZ^-^) (yellow arrowheads).

**Fig. 2:**
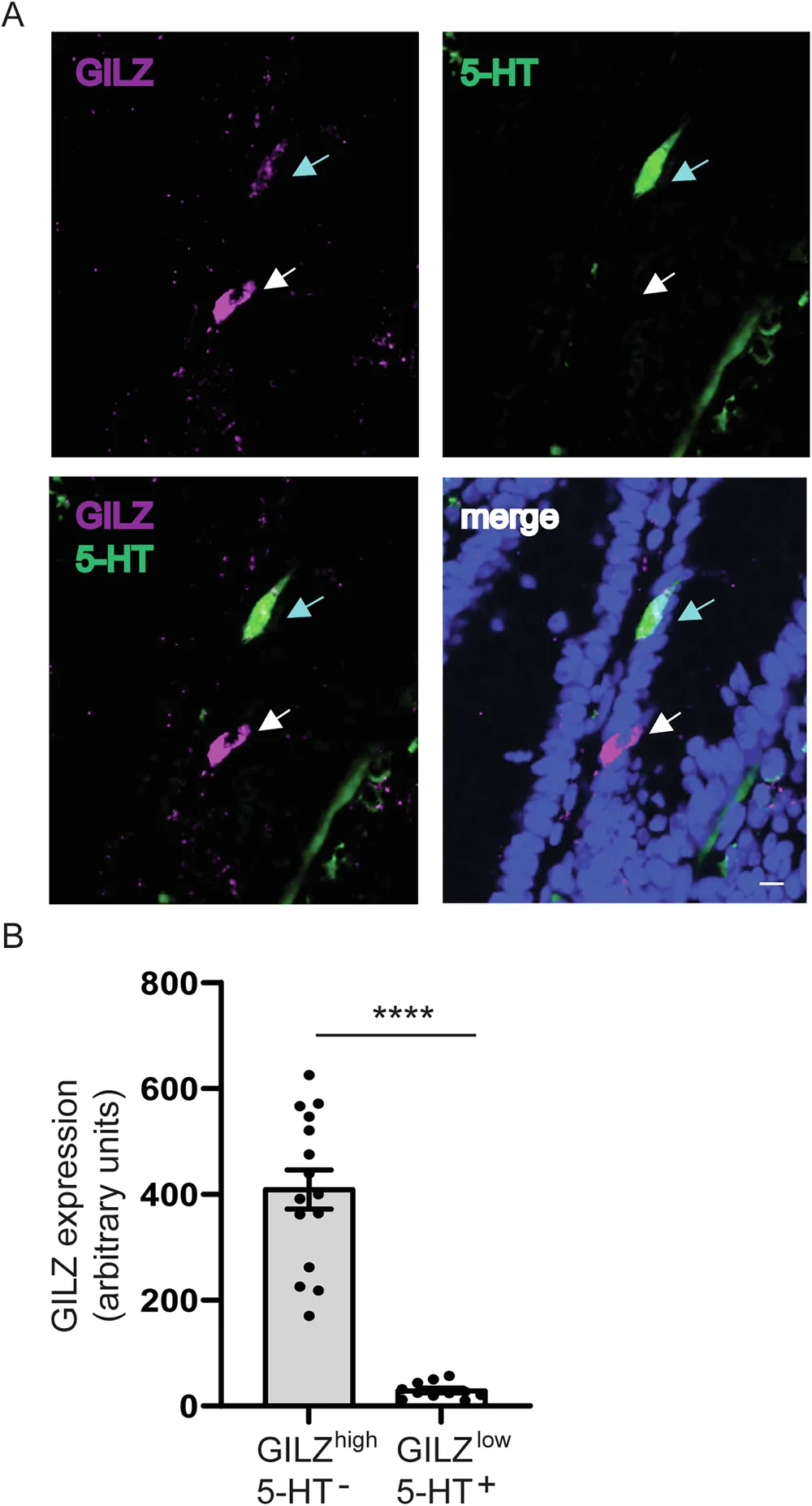
Representative confocal microscopy image of a human colon section with two close EEC. **A** The light blue arrow points to a 5HT^+^ cell expressing low levels of GILZ, while the white arrow indicates a 5HT^-^ cell expressing high levels of GILZ. DAPI staining identifies nuclei. Scale bar: 10 µm. **B** ImageJ quantitative analysis of GILZ expression evaluated in *n* = 15 single cells GILZ^high^/5HT^-^ and *n* = 11 GILZ^low^/5HT^+^. Data are represented as mean ± SEM. ****p < 0.0001.

A recent *t*-distributed stochastic neighbor embedding (*t*-SNE) analysis visualized two distinct clusters of EEC in the colon, the EC and L-cells [19, 41]. The confocal images of Fig. 2A (magnification) and Supplementary Fig. 2 (wide field) show that 5HT-positive (5HT^+^) cells expressed very low or negligible levels of GILZ (light blue arrows, from now on identified as GILZ^low^/5HT^+^), while 5HT^-^ cells expressed high levels of GILZ (white arrows, from now on identified as GILZ^high^/5HT^-^). The amount of GILZ protein in GILZ^high^/5HT^-^ and GILZ^low^/5HT^+^ single cells was quantitated by densitometric analysis resulting by mean 14-fold higher in the former (mean: 409.5 ± 37.1 *vs* 29.2 ± 4.6) (Fig. 2B). Both 5HT^+^ and 5HT^-^ cells are GILZ^+^, but they express very different amount of GILZ protein.

**Fig. 3:**
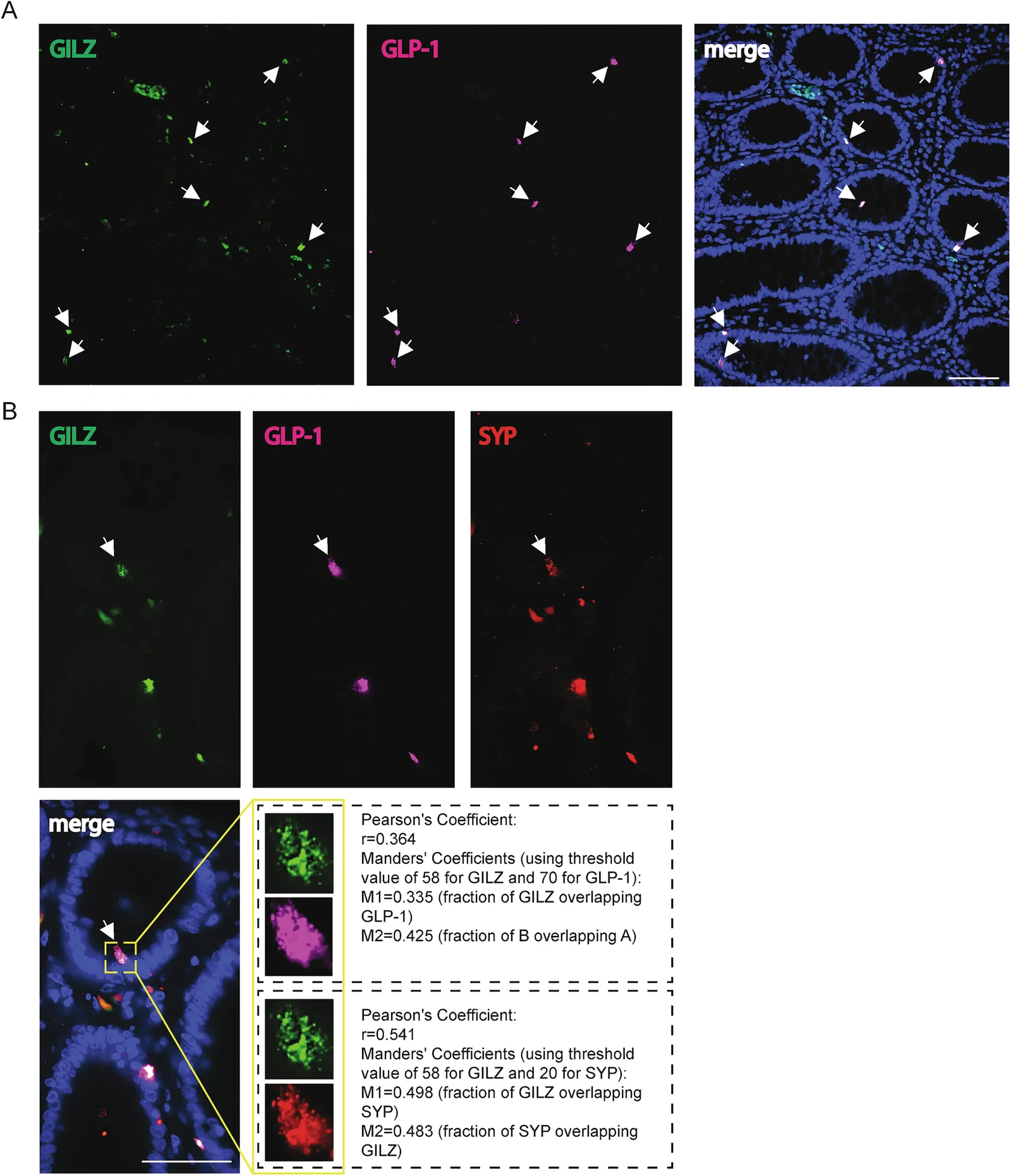
Representative confocal microscopy images of human colon sections with L-cells. **A** GILZ^+^/GLP-1^+^ cells are indicated by arrows. Scale bar: 100 µm. **B** Triple staining with anti-GILZ, anti-GLP-1 and anti-SYP Abs (DAPI identifies nuclei) demonstrate co-localization of the three proteins. Insets show a magnified selected cell with comparative quantification of GILZ-GLP-1 co-localisation (upper box) and GILZ-SYP co-localization (lower box), analysed with *JACop* ImageJ plugin. Pearson’s coefficient value and Manders coefficients values are indicated for GILZ-GLP-1 co-localization (top panel) and GILZ-SYP co-localization (bottom panel) in dashed boxes. Scale bar: 50 µm.

To further characterize the phenotype of GILZ^high^/5HT^-^ EEC, which according to recent work might be referred to as L-cells [24, 41], we co-stained colon sections with anti-GLP-1 and anti-GILZ antibodies (Abs). The images in Fig. 3A show that indeed GILZ^high^ cells were positive for GLP-1, therefore they were identified as GILZ^+^/GLP-1^+^ L-cells (white arrows). Furthermore, a triple staining using anti-GILZ, anti-GLP-1 and anti-SYP Abs showed that GILZ exhibited a granular expression pattern, suggesting GILZ as a secreted protein by L-cells, similarly to GLP-1 (Fig. 3B, arrowhead). Overlay images showed GILZ-GLP-1 partial co-localization. The extent of co-localization was calculated by ImageJ software with *JACoP ImageJ plugin*, as described in [42]. The Manders’ coefficient of 0.335 means partial co-localization of GILZ with GLP-1 (about 30%), suggesting that the majority of GILZ- and GLP-1-containing cytoplasmic granules were distinct, thus implying independent vesicular secretion (Fig. 3B, inset). Similarly, the overlay images of GILZ and SYP were analysed. The Manders’ coefficient of 0.498 means partial co-localization of GILZ with SYP, but more extended than with GLP-1 (about 50%). An in depth analysis of this triple staining revealed that the majority of L-cells was GILZ^+^/GLP-1^+^ (white arrows), whereas a small subset was GILZ^-^/GLP-1^+^ (yellow arrows); conversely, another small proportion of EEC resulted GILZ^-^/GLP-1^-^ (light blue arrows), only exhibiting SYP expression, indicating that not all SYP^+^ cells are L-cells and not all L-cells express GILZ (Supplementary Fig. 3). In a triple co-staining of GILZ, GLP-1 and 5-HT, all these findings were further confirmed (Supplementary Fig. 4).

We next assessed the frequency of GILZ^+^/GLP-1^+^ L-cells over the total GLP-1^+^ cell population in ileal and colorectal biopsies from healthy individuals. Supplementary Fig. 5 shows an increasing gradient of cell density across the small and large bowel in healthy colonic mucosa.

### GILZ is expressed in progenitors and mature EEC

**Fig. 4:**
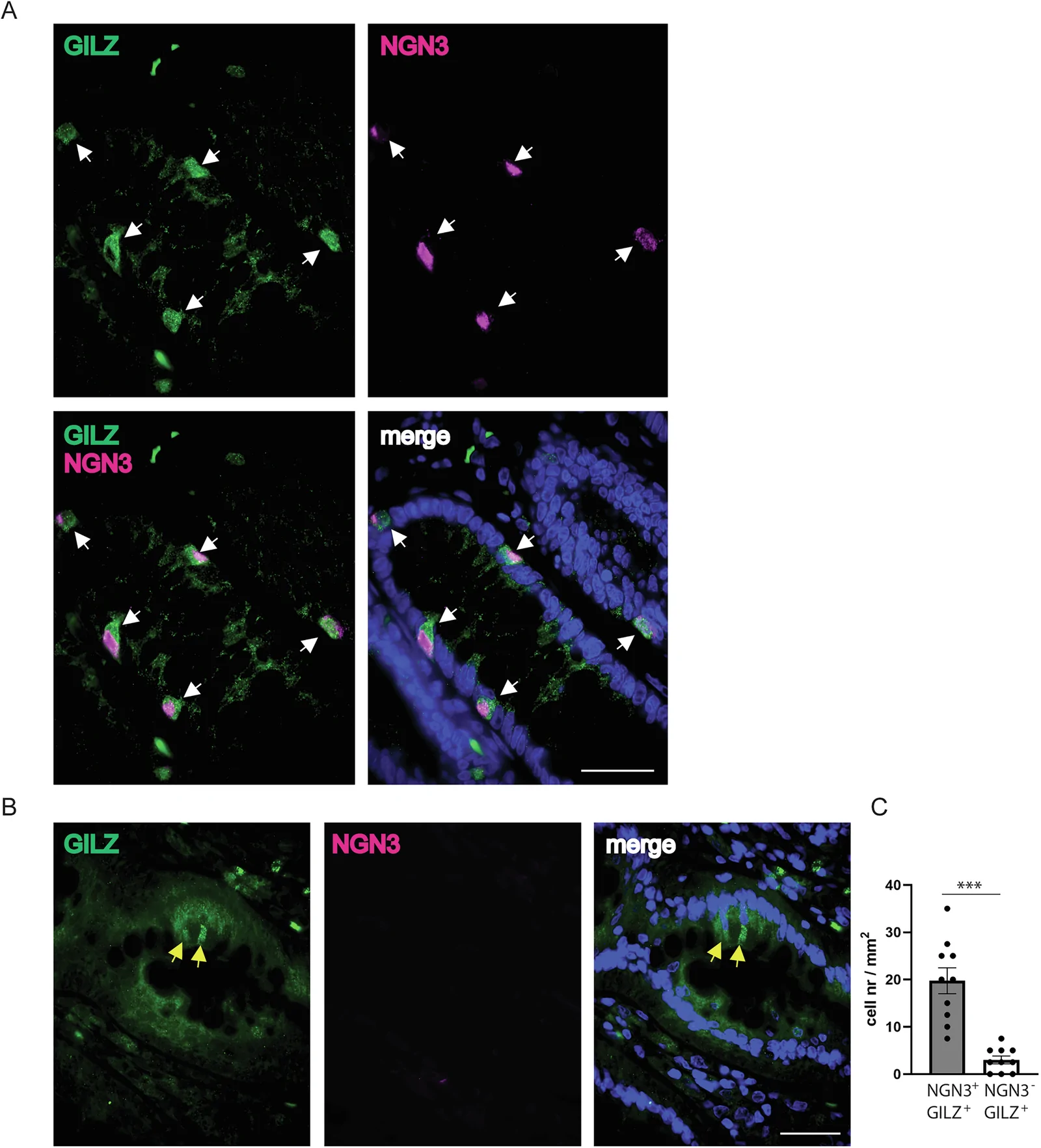
Representative confocal microscopy images of human colon sections. **A** Double staining with anti-GILZ and anti-Neurogenin 3 Abs: white arrowheads indicate double positive (GILZ^+^/NGN3^+^_)_)EEC progenitors. Scale bar: 50 µm. **B** Yellow arrowheads indicate GILZ^+^ cells with a typical secretion pattern, not expressing NGN3. Nuclei were counterstained with DAPI. Scale bar: 50 µm. **C** The graph shows the number of specific cells per mm^2^, as mean ± SEM. ***p < 0.001.

Expression of preproglucagon (GCG), precursor of GLP-1 and GLP-2, is known to increase upward into the crypt, according to the stage of EEC differentiation [25]. The observation that GILZ^+^/GLP-1^+^ cells were scattered along the crypts (Supplementary Fig. 6) suggested that GILZ might be expressed at different stages of EEC development. To this end, we provided an assessment of GILZ expression in subsequent stages of EEC maturation, by co-staining colonic samples with anti-GILZ and anti-Neurogenin 3 (NGN3) Abs. NGN3 is a transcription factor that drives EEC commitment and is considered a marker of EEC progenitors [43, 44]. The majority of NGN3^+^ cells co-stained with GILZ (NGN3^+^GILZ^+^), whereas only a few cells were NGN3^-^GILZ^+^ (Fig. 4A-C), indicating that GILZ is expressed from EEC precursors to mature cells into the crypt. In addition, GILZ-NGN3 co-staining in the majority of cells suggested that GILZ expression can be referred to as an early event in EEC maturation.

**Fig. 5:**
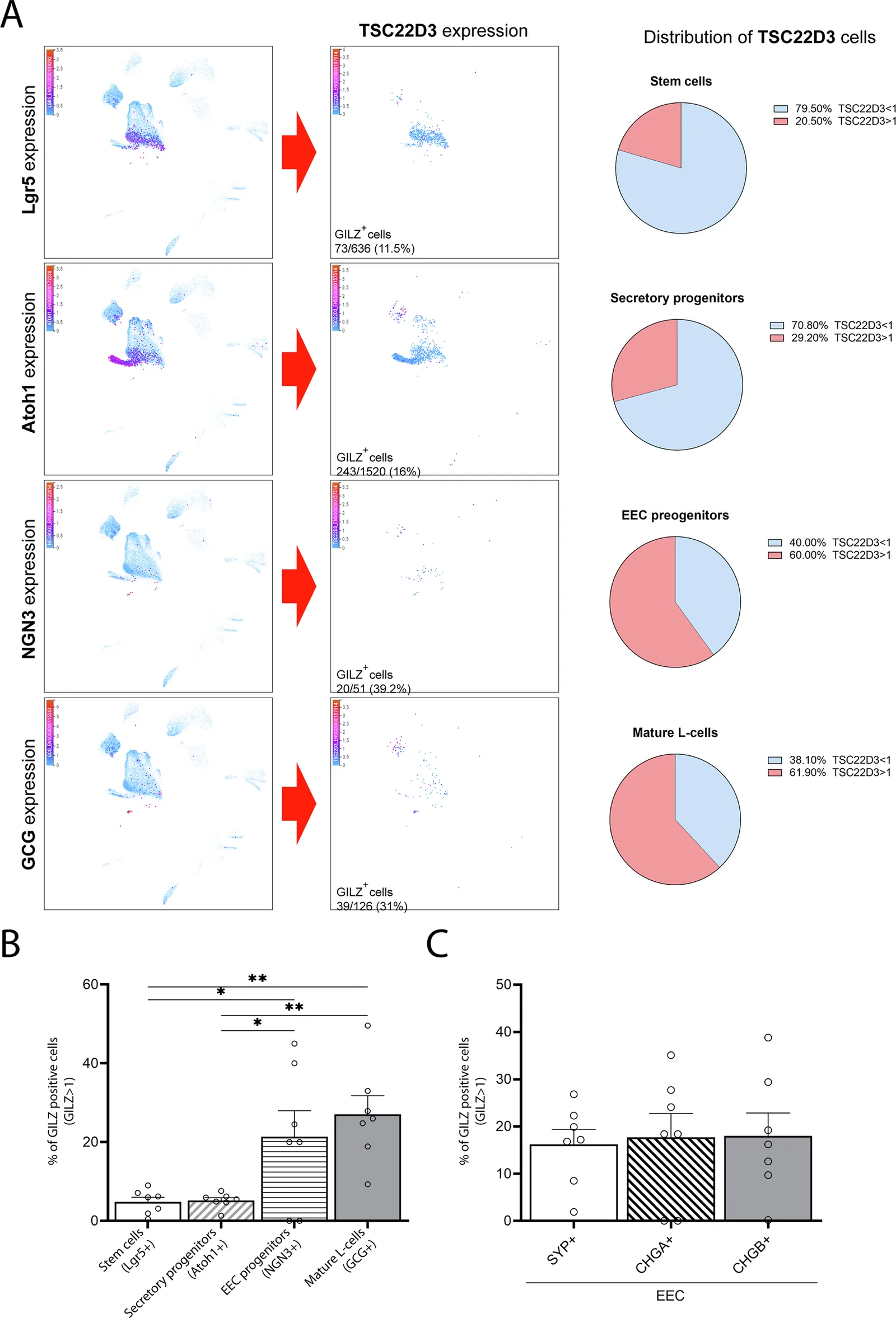
Analysis of single-cell RNA sequencing (scRNA-seq) data from human colon samples retrieved from the Human Cell Atlas (HCA) Data Portal. **A** UMAP projections of colonic single cells showing the expression levels of markers for the stages of EEC development (left column; color scale from light blue to red). The central column shows GILZ (*TSC22D3*) expression (GILZ > 0) in cells positive for each corresponding marker, while the pie charts (right column) summarize the proportion of cells expressing high levels of GILZ (cut-off value 1, GILZ > 1 red) and low levels of GILZ (GILZ < 1, light blue) within each population. **B** The histogram shows the percentage of GILZ > 1 cells across seven individual donors during different stages of EEC cell maturation. **C** The histogram shows the percentage of GILZ > 1 cells across different mature EEC subpopulations. Data are presented as mean ± SEM; individual donors are represented by circles. *p < 0.05; **p < 0.01.

To further analyse GILZ expression in human specimens, with a particular focus on EEC, avoiding the limitation of a protein-threshold level in the immunofluorescence technique, we used the single cell (sc)RNAseq analysis of publicly available dataset [45]. We identified the stages of EEC maturation by the expression of temporally activated transcription factors as reported in *Guo* et al. [43]. Hence, EEC were sub-classified into four populations, including stem cells, secretory progenitors, EEC progenitors, and mature L-cells, which were identified by expressing Lgr5, Atoh1, NGN3, and GCG, respectively. The percentage of cells expressing GILZ gene (TSC22D3) was evaluated in healthy subjects (*n* = 7) (Fig. 5A). An increase in GILZ expression was found from EEC progenitors to mature L-cells (Fig. 5B). In mature EEC, identified by SYP, ChgA and ChgB expression, GILZ^+^ cells were less than 20%, regardless the marker of neuroendocrine differentiation (Fig. 5C).

### GILZ is dysregulated in EEC of UC patients

**Fig. 6:**
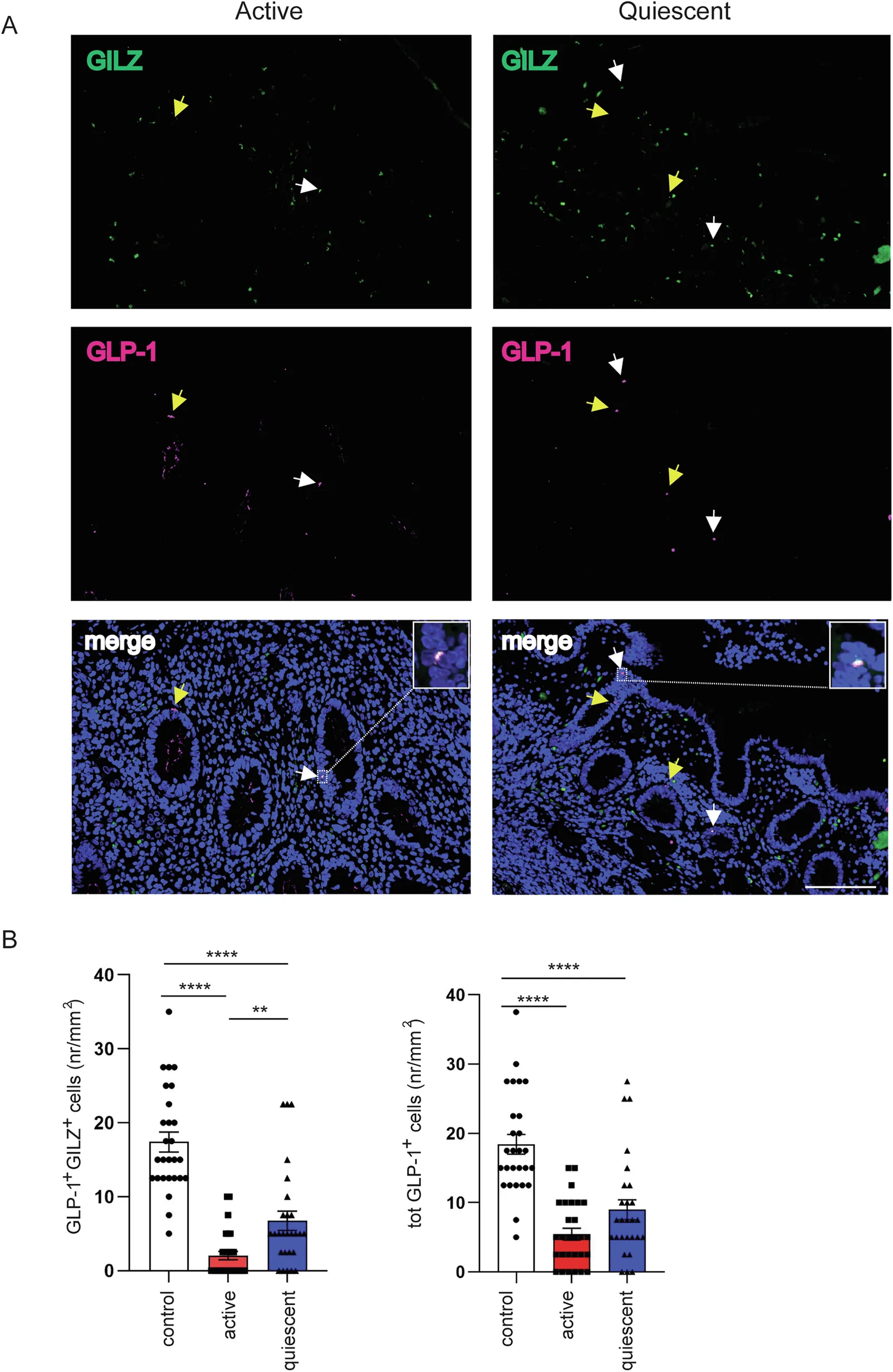
Representative confocal microscopy images of biopsies from UC patients. **A** GILZ and GLP-1 co-staining in both active and quiescent UC. GLP-1^+^/GILZ^+^ L-cells and GLP-1^+^ cells are indicated by white and yellow arrows, respectively. An enlarged image of one selected cell is provided in the inset. **B** Graphs show the number of GLP-1^+^/GILZ^+^ cells (left graph) and total GLP-1^+^ cells (right graph) in healthy controls, active UC and quiescent UC. Scale bar: 100 µm. Data are presented as mean ± SEM. **p < 0.01, ****p < 0.0001.

We next analysed the distribution of GILZ^+^ L-cells in mucosal samples from UC patients (*n* = 84). Diagnostic criteria for active UC were met in 42 biopsies. The remaining 42 biopsies showed hyperplastic changes without neutrophil mucosal infiltration, and were referred to as quiescent UC. SYP^+^ cells were found to be significantly decreased in active UC (5.1 ± 0.9/ mm^2^), as compared to quiescent UC (33.8 ± 4.3/ mm^2^). Likewise, ChgA^+^ EEC were decreased in active UC (9.4 ± 0.8/ mm^2^) as compared to quiescent UC (34.3 ± 3.5/ mm^2^) (Supplementary Fig. 7). Figure 6A shows immunofluorescence images of biopsies from both active and quiescent UC, with scanty both GILZ^+^/GLP-1^+^ L-cells (white arrows) and total GLP-1^+^ L-cells (white and yellow arrows), compared to samples from healthy individuals (Supplementary Figs. 8A and 3A). We assessed the number of L-cells per mm^2^, including the analysis of sections from healthy individuals (Ctrl, Supplementary Fig. 3 and Supplementary Fig. 8A). GILZ^+^/GLP-1^+^ (2.0 ± 0.6 cells / mm^2^) and total GLP-1^+^ cells (5.4 ± 0.9 cells / mm^2^) were significantly reduced as compared to controls (17.4 ± 1.3 cells / mm^2^ and 18.4 ± 1.4 cells / mm^2^, respectively) in active UC (Fig. 6B). Interestingly, a significant increase of GILZ^+^/GLP-1^+^ was observed in quiescent UC (6.8 ± 1.3 cells / mm^2^), but below the level of controls; conversely, total GLP-1^+^ cells did not increase significantly in quiescent UC (8.9 ± 1.4 cells / mm^2^).

**Fig. 7:**
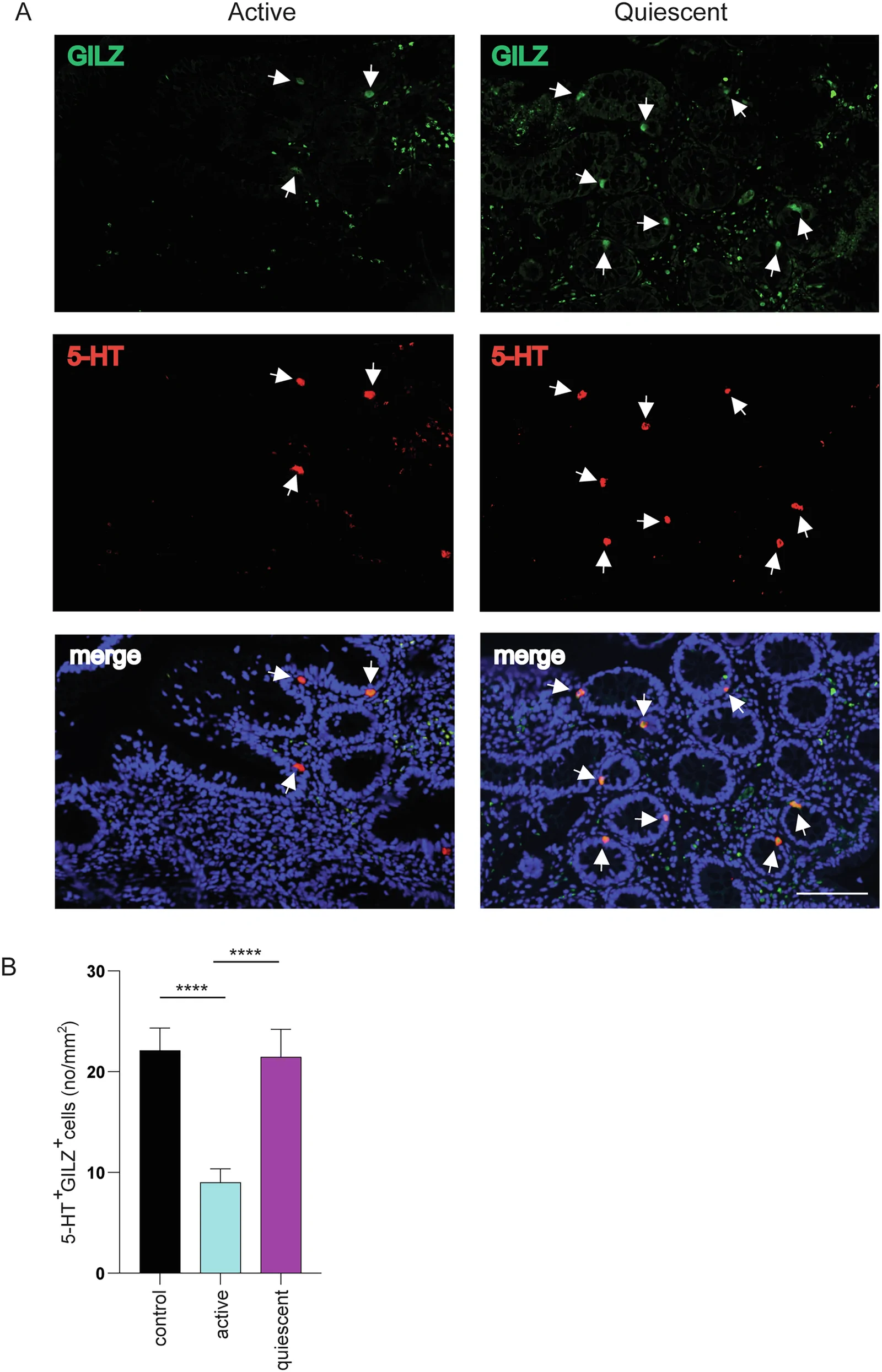
Representative confocal microscopy images of biopsies from UC patients. **A** GILZ and 5HT staining in both active and quiescent UC. Individual cells are indicated by arrows. **B** The graph shows the number of 5HT^+^/GILZ^+^ cells in healthy colons, active UC and quiescent UC. Scale bar: 100 µm. Data are presented as mean ± SEM ****p < 0.0001.

We next analysed the distribution of GILZ^low^/5HT^+^ EC in UC. Immunofluorescence images in Fig. 7A showed a significant reduction in GILZ^low^/5HT^+^ cells (9.0 ± 1.3 cells / mm^2^) in active UC as compared to control (22.1 ± 2.2 cells / mm^2^, Supplementary Fig. 8B and Supplementary Fig. 2), which was completely restored in quiescent UC (21.5 ± 2.8 cells / mm^2^) (Fig. 7B).

### GILZ in L-cells exerts an anti-inflammatory role on inflamed epithelial cells

**Fig. 8:**
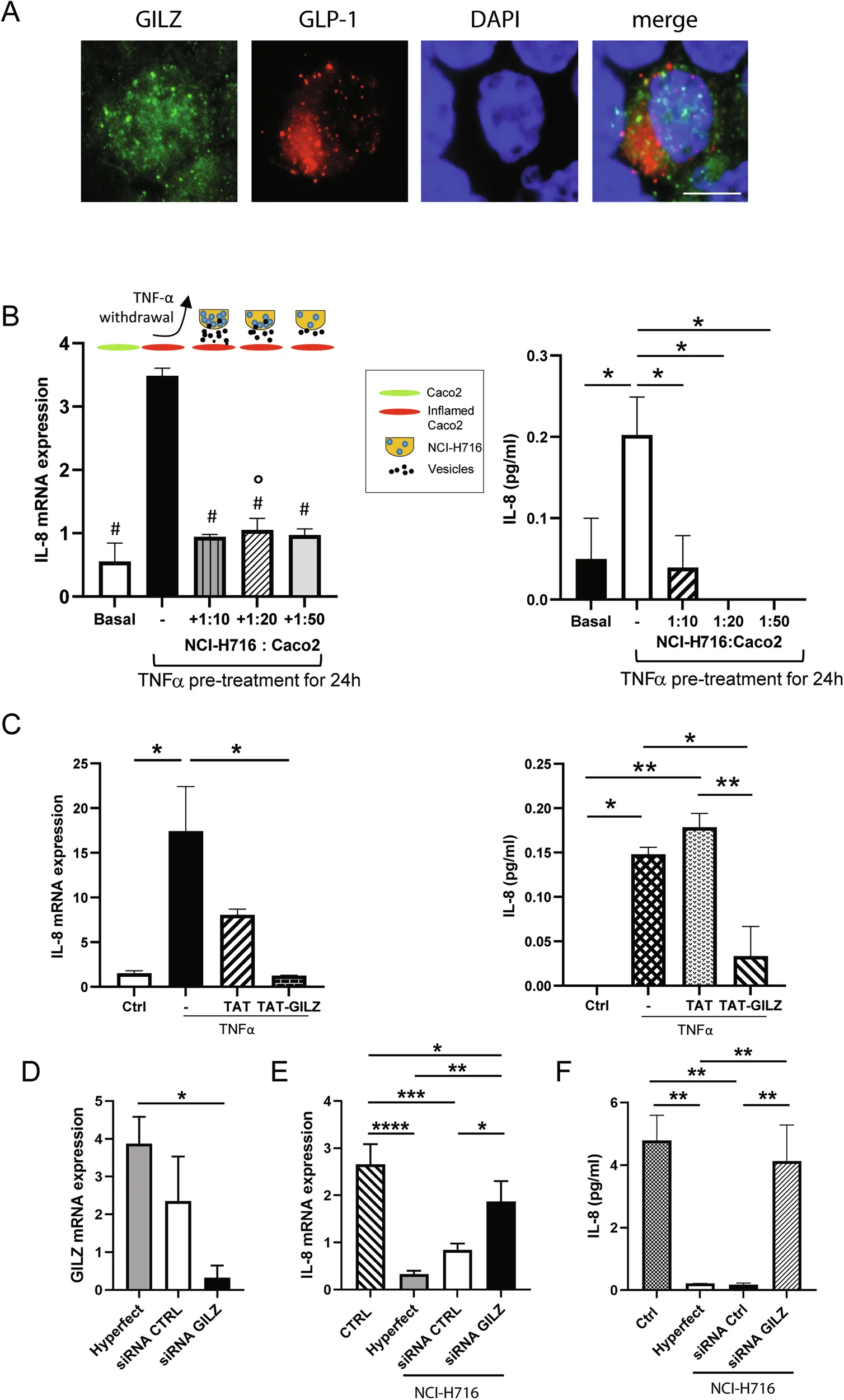
In vitro activity of NCI-H716-derived vesicles and TAT-GILZ on epithelial Caco2 cells. **A** Immunofluorescence images showing co-staining of GILZ (green) and GLP-1 (red) in NCI-H716. Nuclei were counterstained with DAPI. Scale bar: 10 µm. **B** In vitro co-culture of NCI-H716 and Caco2 cells in transwell plates. Caco2 cells were exposed to TNFα for 24 h before apical addition of NCI-H716 at the indicated ratios. After 24 h of co-culture, mRNA was extracted from Caco2 and IL-8 expression was measured by RT-qPCR. The results are the mean ± SEM of two independent experiments, each run in triplicate. # *vs* control (black column); ° *vs* basal. #*p* < 0.0001; °*p* < 0.05. ELISA quantitative evaluation of IL-8 released in the medium in the 48h-co-cultures. **C** Caco2 cells were treated with recombinant TAT-GILZ or related-control (TAT) after 24 h exposure to TNFα. IL-8 expression was measured both by RT-qPCR after 24 h and ELISA after 48 h. The results are presented as the mean ± SE of two independent experiments, each run in triplicate. **D** In vitro co-culture of NCI-H716 and Caco2 cells in transwell plates. Caco2 cells were exposed to TNFα for 24 h before apical addition of silenced NCI-H716 at the 1:10 ratio (NCI-H716 : Caco2). NCI-H716 were silenced 24 h earlier with either siRNA CTRL (HS positive control) or siRNA GILZ (siRNA TSC22D3). A further vehicle control with only the transfection reagent was added (Hyperfect). After 24 h of co-culture, mRNA was extracted from both silenced NCI-H716 and Caco2 cells to measure GILZ and IL-8 expression (**E**), respectively, by RT-qPCR. **F** ELISA assay to quantify the IL-8 release in the culture medium after 48 h co-culture. The results are the mean ± SEM of two independent experiments, each run in duplicate. *p < 0.05, **p < 0.01, ***p < 0.001, ****p < 0.0001.

The function of GILZ in L-cells was investigated in vitro, using the human L-cell line NCI-H716. We first assessed GILZ expression in these cells by immunofluorescence co-staining with GLP-1. The image in Fig. 8A shows GILZ expressed with a cytoplasmic granular/vesicular pattern, suggesting secretory functions via granules or vesicles delivery, as previously described for GLP-1 [46]. To investigate whether vesicle delivery could exert an anti-inflammatory effect, we set up an in vitro co-culture of NCI-H716 cells and gut epithelial cell line Caco2 in transwell plates, to hinder any cell-to-cell contact between the two cell lines. Caco2 cells were exposed to TNFα as an inflammatory stimulus, for 24 h. After TNFα withdrawal, NCI-H716 cells were added to inserts in Caco2 culture wells. IL-8 induction in Caco2 was measured as an index of cell inflammatory response, since IL-8 is a chemokine capable to attract neutrophils in the mucosa, starting trigger of inflammation in UC. Figure 8B shows that NCI-H716-secreted content was able to reduce IL-8 mRNA expression after 24 h, independently of the amount of added NCI-H716 cell number. ELISA measurement of the released IL-8 confirmed the reduction after 48 h (Fig. 8B). These results suggest that vesicles released by NCI-H716 can reduce inflammation, and GILZ in these granules could contribute to this anti-inflammatory effect. To directly test this hypothesis in another set of experiments, we treated Caco2 cells with TAT-GILZ recombinant protein, which can directly enter the cells. After exposure of Caco2 cells to TNFα for 24 h, the addition of TAT-GILZ protein significantly reduced the expression of IL-8, as measured by RT-qPCR. Accordingly, IL-8 released in the culture medium was reduced 48 h later (Fig. 8C). To further substantiate the contribution of GILZ to the anti-inflammatory effect of L-cell-derived products, GILZ expression was silenced in NCI-H716 cells. The cell cycle distribution of untreated and Hyperfect-treated NCI-H716 cells (controls) was first evaluated at 24 and 48 h post-silencing. No significant differences were observed in the proportion of cells in G0/G1, S or G2/M phases between untreated and Hyperfect-treated cells (Supplementary Fig. 9A). Accordingly, subsequent analyses compared silenced groups to Hyperfect-treated cells. Twenty-four hours after silencing, NCI-H716 cells were seeded into apical inserts of transwell-plates and co-cultured with TNFα pre-activated Caco2 cells under the same experimental conditions described above. After additional 24 h, cell cycle distribution was assessed in Hyperfect, siRNA CTRL and siRNA GILZ groups to confirm the expected reduction of cell proliferation in the positive control (siRNA CTRL). As shown in Supplementary fig. 9A, siRNA CTRL significantly reduced cell cycle progression compared to Hyperfect- and siRNA GILZ-treated cells, indicating effective silencing of pro-survival genes (detailed in Materials and Methods). Total RNA was isolated from apical inserts from Hyperfect, siRNA CTRL and siRNA GILZ groups (48 h post-silencing) and GILZ expression was quantified. A schematic representation of the experimental workflow is provided in Supplementary fig. 9C. As shown in Fig. 8D, GILZ expression was significantly reduced in siRNA GILZ cells compared with controls. To determine the effect of GILZ silencing on IL-8 production, IL-8 expression was measured in Caco2 cells 24 h after co-culture with silenced NCI-H716 cells. Consistent with the results shown in Fig. 8B, IL-8 expression was significantly suppressed in Caco2 cells co-cultured with Hyperfect- and siRNA CTRL-treated cells. In contrast, IL-8 levels were significantly increased in Caco2 cells co-cultured with siRNA GILZ-treated cells (Fig. 8E). However, IL-8 expression in this group was not completely restored to the levels of TNFα-pre-treated control cells, suggesting that GILZ contributes to the anti-inflammatory effect of L-cell-secreted products, although it is not the sole mediator of this activity. IL-8 protein concentration in the culture supernatants was quantified 48 h later by ELISA. As shown in Fig. 8F, a significant reduction in IL-8 release was observed in control groups (Hyperfect and siRNA Ctrl), whereas IL-8 levels resulted in a marked increase in the siRNA GILZ, reaching values comparable to those detected in TNFα-pre-treated Ctrl, suggesting an accumulation of secreted IL-8 in the culture medium.

## Discussion

Previous studies in both animal models and humans have demonstrated the role of GILZ as an anti-inflammatory protein implicated in IBD, affecting immune responses as well as goblet cell functions [2, 4–7, 11, 47–49]. In the present study, we identified high GILZ expression levels in L-cells, a specific subtype of EEC. In these cells, GILZ was partially co-expressed with both GLP-1, the major incretin released by colonic L-cells, and synaptophysin, a membrane glycoprotein expressed in the synaptic-like microvesicles of EEC [50, 51]. Importantly, GILZ expression was detected as an early event during EEC differentiation, being detected in both precursors and mature cells. This observation supports a potential functional role of GILZ throughout the entire EEC lifecycle. Consistently, GILZ^+^/GLP-1^+^ cells were distributed along the length of the colonic crypts, indicating that GILZ expression occurs across subsequent developmental stages of L-cells. Transcriptomic scRNAseq analysis further confirmed our findings, showing elevated GILZ expression in mature secretory cells, particularly within the EEC population.

A deep examination of GILZ expression in human gut samples by immunofluorescence analysis revealed a progressive increase in GILZ-expressing L-cells along the gut, displaying an upward trend from the ileum to the rectum. This spatial distribution closely paralleled the pattern observed for GLP-1 expression, suggesting a potential coordinated secretion of GILZ and GLP-1 in L-cells. This observation is consistent with previous evidence demonstrating that L-cell density increases distally along the human intestine toward the rectum [25]. In active UC, we observed a significant reduction of GLP-1^+^/GILZ^+^ cells and total GLP-1^+^ cells compared to controls. Notably, GLP-1^+^/GILZ^+^ cells were restored in quiescent UC, even though at a lesser degree as compared to controls, whereas no differences were observed in total GLP-1^+^ cell number, suggesting GILZ is important for the restoration of L-cells. The intestinal environment, which is highly sensitive to inflammation, number, distribution and function of cells may be affected by active disease, pro-inflammatory cytokines and epithelial barrier dysfunction. Hence, varying degrees of epithelial cell restoration can be observed in quiescent UC, including number and function of cells, mostly depending on the duration of the regenerative process and on the magnitude of the acute inflammation [52]. Intriguingly, GLP-1 is synthesized intracellularly and released through secretory vesicles in response to dietary nutrients and it is known to participate in the autocrine/paracrine signaling, which may be disrupted in IBD [53, 54]. In particular, GLP-1 can influence the vesicular release of other molecules, as it contributes to exosome biogenesis [55]. Therefore, the reduced GLP-1 expression may drive the reduction of GILZ levels, which might negatively affect the epithelial repair, as observed in preclinical studies [49]. The loss of GILZ^+^ L-cells in active UC correlates with the inflammatory environment, potentially promoting the impairment of gut homeostasis. This finding aligns with our previous observation of GILZ reduction in goblet cells from IBD patients [11]. Supporting this role, the administration of recombinant TAT-GILZ protein has been shown to ameliorate symptoms of spontaneously induced colitis in IL-10-KO mice and other experimental models by modulating either T or B infiltrating lymphocytes (revised in [48]), and by strengthening the mucosal barrier in DSS-induced colitis [49]. Together, these findings from both animal and human studies support the concept that GILZ contributes to maintain intestinal homeostasis.

Intriguingly, colonic L-cells showed a distinct cytoplasmic localization of GILZ, resembling vesicle-like structures. This observation suggests GILZ may be implicated in vesicular trafficking, similar to other L-cell products [56]. Although these GILZ-containing vesicles have not yet been fully characterized, their nature warrants further investigation.

Our in vitro experiments aimed at identifying GILZ function in L-cells. Vesicles secreted by NCI-H716 L-cells were found to downregulate IL-8 expression in co-cultured Caco2 epithelial cells, suggesting that GILZ, together with GLP-1, contributes to the suppression of this key pro-inflammatory chemokine. IL-8, produced by epithelial cells after mucosal barrier damage, plays a central role in recruiting neutrophils to the mucosa. While neutrophil infiltration is essential for pathogen clearance, excessive accumulation can lead to transepithelial migration, crypt architectural disruption, and exacerbation of inflammation in IBD [57, 58]. By reducing IL-8 levels, GILZ release from L-cells may contribute to reduce neutrophil-driven mucosal injury and inflammation. This function is further supported by our demonstration that the recombinant TAT-GILZ protein directly suppresses IL-8 production in epithelial cells and GILZ silencing reduces the ability of secreted L-cell proteins to suppress IL-8 produced by inflamed epithelial cells. Interestingly, GLP-1 also exerts anti-inflammatory effects, since previous studies demonstrated that GLP-1 alleviates DSS-induced colitis in murine models by reducing inflammation [59, 60]. This function may explain the fact that GILZ contributes to prevention of the release of IL-8, but is not the sole protein to exert this effects. Interestingly, accumulated IL-8 protein in the culture supernatant of GILZ-silenced cells reached the control levels after 48 h. Our results add data to the known extracellular vesicle-mediated immune responses in the gut [61]. A limit of this study is the unknown mechanism of GILZ-mediated IL-8 suppression, which does not relies on NF-κB inhibition (data not shown) but on other factors, which deserve future studies. Furthermore, GLP-1 is secreted in response to glucose intake, sustaining a gluco-regulatory system that acts by increasing insulin and suppressing glucagon secretion [24, 62]. Impaired GLP-1 secretion can lead to clinical susceptibility to abnormal glucose metabolism, supporting the clinical association between IBD and diabetes [63–65]. Our findings open future perspectives toward the combined restoration of GLP-1 and GILZ as a promising therapeutic strategy to dampen inflammation and promote mucosal healing. Particularly, the role of goblet cells as cellular target of the anti-inflammatory effects of L-cells secretion suggests that even the barrier functions can be improved in UC patients.

A distinct scenario was observed for EC 5HT^+^ cells. In these cells, GILZ expression was consistently low, resulting unaffected by disease activity. Interestingly, 5HT^+^ EC cell count decreased significantly during active UC, but was completely restored in quiescent UC. However, previous studies investigating mucosal 5-HT levels and EC cell dynamics in IBD have yielded conflicting results: some studies described an increase in EC cell numbers in UC patients and experimental models of colitis [66], whereas others reported a decrease [67–69]. Despite these discrepancies, it is evident that EC cell abundance and 5-HT levels are altered in active UC, and our data support the observations about the decrease in EC numbers. 5HT^+^/GILZ^+^ cells were found to be completely restored in the quiescence, but this effect may not depend on GILZ function, due to its very low expression levels. The specific contribution of GILZ to EC biology still remains unclear and warrants further investigation.

## Conclusions

Our study identifies GILZ as a novel EEC product, expressed in L-cells. GILZ can potentially facilitate the crosstalk between epithelial cells and immune system, contributing to colon homeostasis. Altered GILZ expression in L-cells may be associated with either the onset or relapse in UC. Specifically, the loss of GILZ^+^ L-cells during active UC, together with impaired secretion of both GILZ and GLP-1, could further impair the barrier dysfunction of the gut mucosa, compromise responsiveness to conventional therapies, and contribute to extra-intestinal IBD-related manifestations, including diabetes. Restoring GILZ expression in L-cells, particularly in combination with approaches that trigger GLP-1 secretion or mimic its actions, may represent a promising pharmacological approach in UC patients.

## Materials and methods

### Immunohistochemistry and immunofluorescence

Biopsy samples from healthy colonic mucosa and UC patients were retrospectively analysed, according to the Ethics Committee Spedali Civili, Brescia, ID no NP 4811. Four μm-thick slides were cut from formalin-fixed paraffin-embedded (FFPE) specimens, and stained with Hematoxylin-Eosin dye. Specimens from UC patients were evaluated for architectural distortion, mucin depletion, occurrence of basal plasma cells, and disease activity by experienced GI pathologists. Neutrophil infiltration, cryptitis and crypt abscesses, erosion and ulcerations were referred to active UC, and were evaluated in all the samples. Overall, 120 mucosal pinch biopsies were examined, including 84 samples from UC patients and 36 samples from healthy donors.

For immunofluorescence assays, FFPE specimens were cut and deparaffinized. After rehydration, antigen retrival was performed according to the specific Ab. Immunostaining was performed with the following Abs: anti-GILZ (ab197987, Abcam, Cambridge, UK), anti-5-HT (ab6336, Abcam), anti-GLP-1 (ab26278, Abcam) anti-ChrgA (sc-271738, Santa Cruz, Dallas, Texas, USA), anti-Neurog3 (sc-376607, Santa Cruz) and anti-Syp (OSS00058W-100UL, Thermo Fisher Scientific, Waltham, MA, USA). The anti-GILZ antibody has been granted as highly specific by the manufacturer. All the Abs were incubated overnight at 4°C as previously described [11]. Secondary Abs (Alexafluor 488, Alexafluor 555, Alexafluor 640, Thermo Fisher Scientific, Waltham, MA, USA) were added the following day and incubated for 1 h at room temperature (anti-rabbit Ab for the detection of GILZ, anti-mouse Ab for the detection of GLP-1 Ab and ChrgA, anti-rat Ab for the detection of 5-HT and anti-sheep Ab for the detection of Syp). An example of secondary Ab staining is shown in Supplementary Fig. 10. The slides were then washed with PBS 0.1% TWEEN 3 times for 5 minutes and then stained with DAPI (D9542, Sigma-Aldrich, Milan, Italy) to detect the nuclei.

All slides were analysed using Eclipse Ti microscope equipped with Confocal Spinning Disk. In healthy specimens and UC biopsies, the fields (high power field –HPF-, 20x magnification, area = 0.4 µm^2^) were randomly chosen; stained cells were counted by two independent researchers in a blinded manner. To statistically determine the number of stained cells in UC patients, 27 HPF from 10 healthy samples, 30 HPF from 10 active UC patients, and 28 HPF from 7 quiescent UC patients were assessed for GLP-1 total and GILZ-GLP-1 co-expression; 18 HPF from 7 healthy samples, 32 HPF from 6 active UC patients, and 24 HPF from 5 quiescent UC patients were assessed for 5HT-GILZ co-expression.

Densitometric analysis of fluorescence in single cells was carried out with the software ImageJ. After isolating, thresholding, and subtracting background, fluorescence intensity mean ± SEM in the selected area was calculated by the software. Cells were analysed within the same field, in separate stained samples. Values below 100 arbitrarily identified low GILZ-expressing cells, values above 100 arbitrarily identified high GILZ-expressing cells. A schematic description of the identification of GILZ^low^ and GILZ^high^ expressing cells distinguishing 5HT (EC cells) and GLP-1 cells (L-cells) is reported in Supplementary Fig. 11. Co-localization analyses of GILZ and GLP-1, or GILZ and SYP were performed using the JacoP plug in for ImageJ, as previously described [42, 70].

NCI-H716 cells were cytospun onto glass slides and fixed with 10% formalin. After washing in PBS, cells were blocked with buffer containing 0.1% Triton-X and 1% bovine serum albumin. Cells were then incubated o.n. with goat anti-GILZ (sc-26520, discontinued, Santa Cruz) and anti-GLP-1 (ab26278, Abcam,) Abs. After washing with PBS Tween 0,1%, cells were incubated for 1 h with secondary anti-goat-Alexa 488 and anti-mouse Alexa 555 (Alexafluor, Thermo Fisher Scientific) Abs. After adding DAPI, slides were mounted with cover glass and analyzed using a Zeiss Axioplan fluorescence microscope (Zeiss, Oberkochen, Germany) equipped with a Spot-2 cooled camera (SPOT Imaging Solution, Sterling Heights, MI, USA).

### Cell cultures

Caco2 cells were a kind gift of Prof. Domenico Delfino. Caco2 cells were cultured in Dulbecco’s Modified Eagle Medium (DMEM) containing 10% of fetal bovine serum (FBS), 1% of non-essential amino acids (MEM NEAA), 1% of sodium pyruvate and 50 IU/mL penicillin and 50 μg/mL streptomycin at 37 °C and 5% CO_2_. NCI-H716 cell line was purchased from CLS Cell Lines Service GmbH (Eppelheim, Germany). NCI-H716 were cultured in RPMI 1640 containing 10% of FBS and 50 IU/mL penicillin and 50 μg/mL streptomycin at 37 °C and 5% CO_2_.

### Treatments and co-cultures

On day -2 of the experiment, Caco2 cells were seeded in 12-wells plates (3×10^5^ cell/ml) and incubated for 24 h at 37°C. On day -1, cells were stimulated with 10 ng/ml Tumor Necrosis Factor α (TNFα) (recombinant human- Cell guidance system, Cambridge, UK). After further 24 hours, TNFα was withdrawn by replacing with fresh medium. Corning® Transwell® 12 well plates (pores 0.4 µM diameter) (Merck, cat# CLS3401-48EA, Darmstadt, Germany) were inserted in each well containing Caco2 cells previously exposed to TNFα for 24 h. NCI-H716 were seeded in the apical side of the transwell (inserts) at concentration ratios of 1:10, 1:20, 1:50 (NCI-H716: Caco2). At day 1 and 2 of co-culture, mRNA extraction from Caco2 cells was carried out to evaluate IL-8 expression. For Caco2-NCI-H716 co-culture experiments, NCI-H716 cells were grown in complete DMEM medium for 2 weeks, and GLP-1 and GILZ expression levels were analysed by RT-qPCR (not shown). No significant changes in either proliferation or mRNA expression levels were noticed. TAT-GILZ (5 μg/mL) recombinant protein, a cell-permeable fusion protein, and control TAT peptide (2.5 μg/mL) were used for in vitro experiments [71, 72]. IL-8 release in the culture supernatants was quantified with Human IL-8 Uncoated ELISA kit (Invitrogen, Thermo Fisher Scientific), according to the manufacturer’s instructions.

### RNA interference assay

NCI-H716 cells were seeded in cell culture flasks at 2×10^5 c/w in 24 well-plate, following the manufacturer’s instructions. Positive control HS cell death (siRNA CTRL) and Hyperfect transfection reagent were added to two separate groups as controls (Qiagen, Hilden, Germany). GILZ was transiently knocked down in NCI-H716 cells by siRNA GILZ (TSC22D3) for 24 h and then added to the inserts on transwell plates, in which Caco2 cells had been previously seeded and treated with TNFα for 24 h (see MM for co-culture experiments in transwell). Twenty-four hours later, mRNA from apical (NCI-H716 silenced) and bottom (Caco2) cells was extracted to measure GILZ and IL-8 expression, respectively. Cell cycle analysis was performed in all groups both 24 h and 48 h after silencing, by propidiun iodide staining. Briefly, cells were collected, centrifuged and suspended in propidium iodide-hypotonic solution, and kept 1 h at 4°C. The analysis was conducted on a Becton Dickinson FACScan running LYSIS II software. For a more detailed description of the entire procedure, see the schematic in Supplementary Fig. 9C.

### Quantitative RT-PCR

mRNA was extracted from Caco2 cells using RNeasy Plus Mini Kit (Qiagen, Hilden, Germany). The extracted mRNA was retro-transcribed by QuantiTect Reverse Transcription Kit (Qiagen, Hilden, Germany). Quantitative Real-time PCR (RT-qPCR) was performed in triplicate on QuantStudio 1 Real-Time PCR System (Applied Biosystem, Waltham, MA, USA), following the TaqMan® Gene Expression Assays Protocol to detect GILZ by a FAM™ probe (cat# Hs00929365_m1, Thermo Fisher Scientific, Waltham, MA USA), and the eukaryotic 18S rRNA endogenous control (VIC™/MGB probe, primer limited) (Cat# 4319413E Thermo Fisher Scientific, Waltham, MA, USA), using TaqMan™ Gene Expression Master Mix (Applied Biosystems, Waltham, MA, USA). SYBR™ Green Assay Protocol (Applied Biosystems, Waltham, MA, USA) was used to measure IL-8 (Forward primer 5’ACTCCAAACCTTTCCACCCC3’- Reverse primer 5’TTCTCAGCCCTCTTCAAAAACTTC3’), using human GAPDH (5’GCTCCTCCTGTTCGACAGTCA3’- Reverse primer 5’GCAACAATATCCACTTTACCAG3’) as housekeeping gene. Samples were run in triplicate and two-three distinct experiments were performed in each experimental setting. The method 2^-∆CT^ was used to calculate the relative expression levels.

### Bioinformatics analysis

Publicly available scRNA-seq data from the study by Elmentaite et al. [45] were retrieved from the Human Cell Atlas (HCA) Data Portal (https://data.humancellatlas.org/). Only data corresponding to normal colonic tissue from adult donors were included in the analysis. Data exploration and visualization were performed using the CZ CELLxGENE platform (https://cellxgene.cziscience.com/). This tool was also used to generate Uniform Manifold Approximation and Projection (UMAP) plots and to quantify the number of positive cells for selected markers in each individual donor.

### Statistical analysis

Statistical analyses were performed using Prism 10.4.2 software (GraphPad Software, Boston, MA, USA). Normality was assessed with Kolmogorov–Smirnov test. Pairwise or multiple comparisons of values with normal distribution were carried out using Student’s t test (unpaired), one-sample t test (theoretical mean = 1) and one-way ANOVA. Post hoc tests were conducted where appropriate. Results were considered significant at p < 0.05, and data are reported as mean ± standard error (SEM).

## Supplementary information

Supplementary figure S1

Supplementary figure S2

Supplementary figure S3

Supplementary figure S4

Supplementary figure S5

Supplementary figure S6

Supplementary figure S7

Supplementary figure S8

Supplementary figure S9

Supplementary figure S10

Supplementary figure S11

## Data Availability

The datasets generated during and/or analysed during the current study are available from the corresponding author on reasonable request.
