# Supplementary material for: Glucocorticoid-induced Leucine Zipper (GILZ) is a novel secreted protein by intestinal L-cells and is dysregulated during active ulcerative colitis
Source: Cell Death Discov. 2026 Jun 17;12:348. doi: 10.1038/s41420-026-03204-w (PMC13518978; doi:10.1038/s41420-026-03204-w)
Supplement: Supplementary file 1 [file 41420_2026_3204_MOESM10_ESM.pdf]

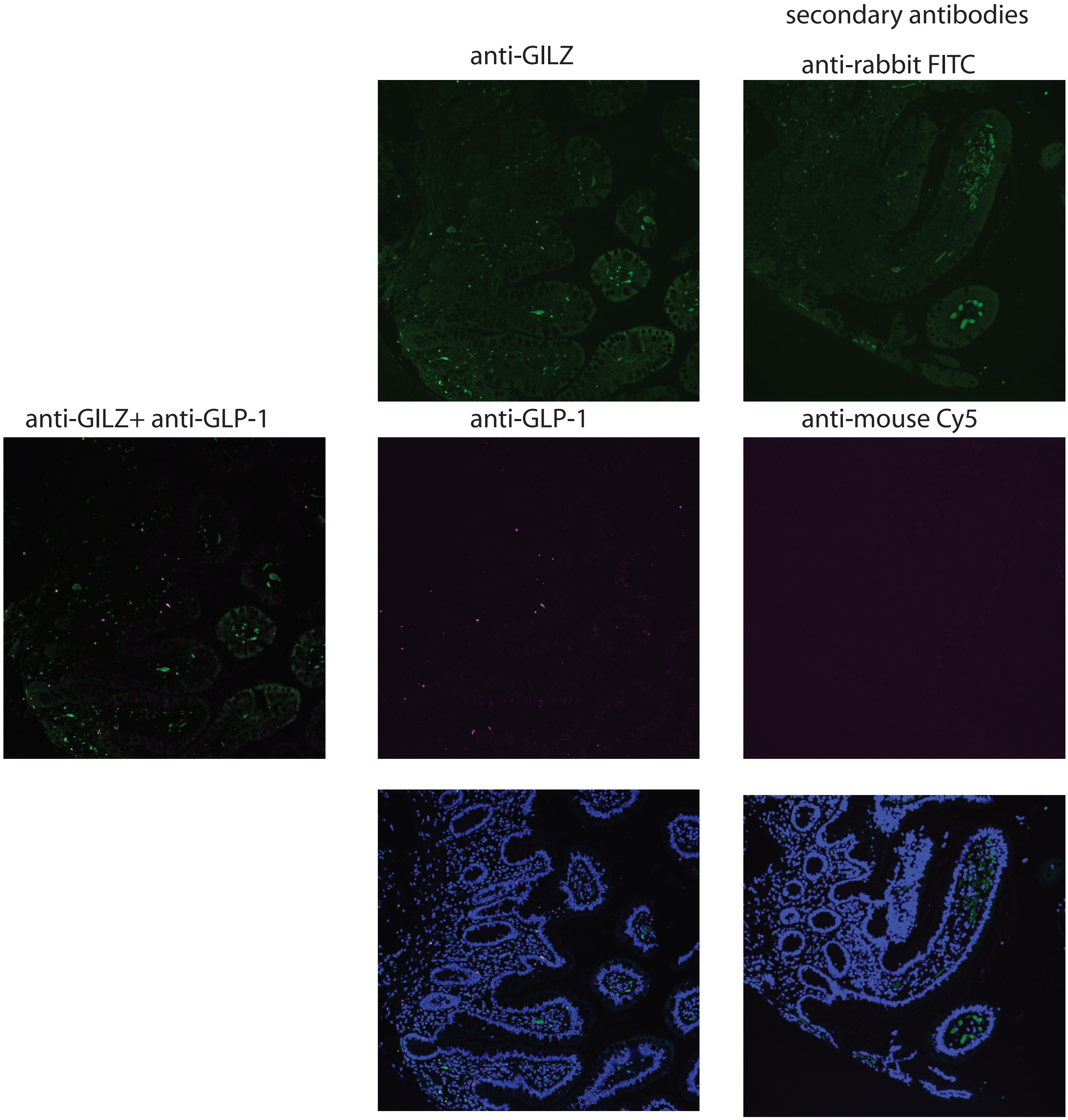

Supplementary Figure 10: confocal images of secondary antibodies in healthy colon tissue sections (right microphotographs). GILZ (green) and GLP-1 (pink) co-stained sections are shown in the left and middle columns. Aspecific immunofluorescence can be observed only in the green channel.
