# Supplementary material for: Glucocorticoid-induced Leucine Zipper (GILZ) is a novel secreted protein by intestinal L-cells and is dysregulated during active ulcerative colitis
Source: Cell Death Discov. 2026 Jun 17;12:348. doi: 10.1038/s41420-026-03204-w (PMC13518978; doi:10.1038/s41420-026-03204-w)
Supplement: Supplementary file 2 [file 41420_2026_3204_MOESM11_ESM.pdf]

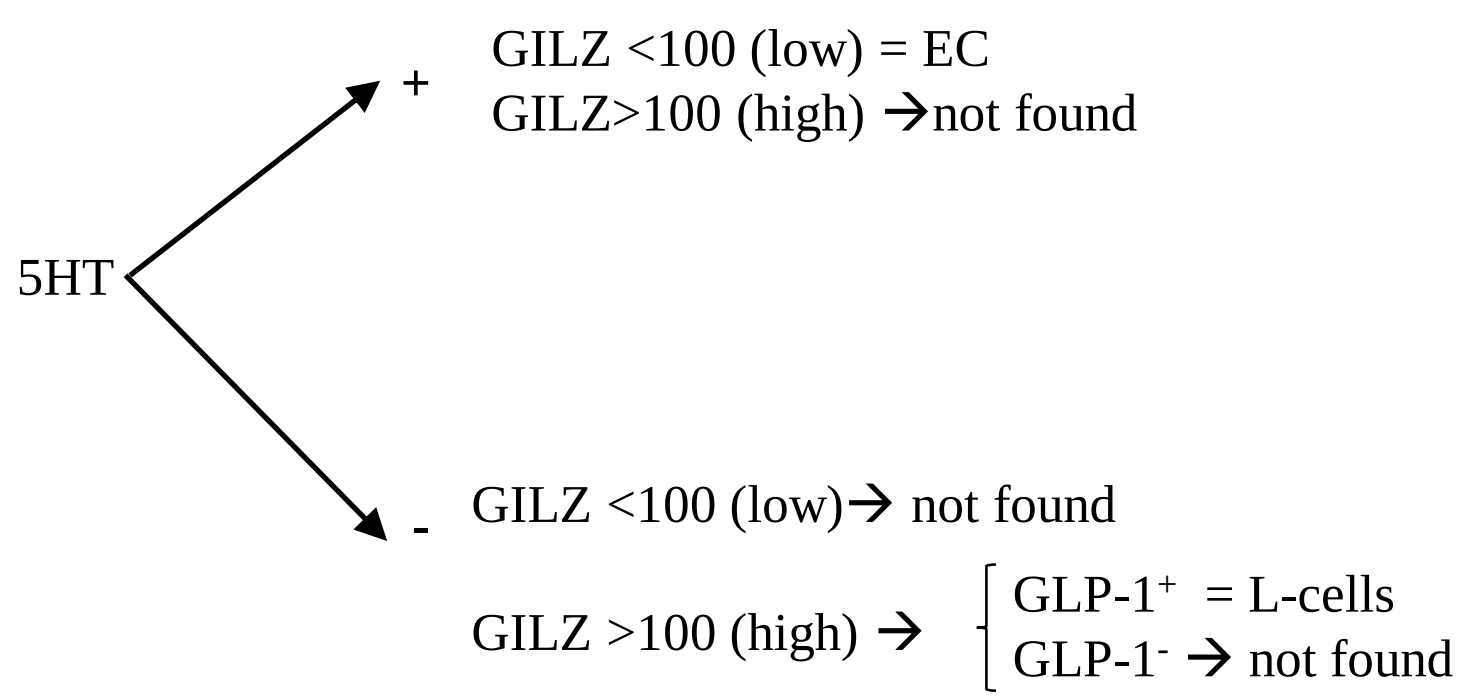

Supplementary Figure 11. A schematic diagram describing the criteria used to identify GILZ^{low} and GILZ^{high} expressing cells: ImageJ was used to quantify the fluorescence intensity of each cell analysed. Values below 100 identified GILZ^{low} cells, whereas values above 100 identified GILZ^{high} cells. GILZ^{low} cells expressed only 5HT, whereas GILZ^{high} cells expressed only GLP-1.
