# Supplementary material for: Glucocorticoid-induced Leucine Zipper (GILZ) is a novel secreted protein by intestinal L-cells and is dysregulated during active ulcerative colitis
Source: Cell Death Discov. 2026 Jun 17;12:348. doi: 10.1038/s41420-026-03204-w (PMC13518978; doi:10.1038/s41420-026-03204-w)
Supplement: Supplementary file 3 [file 41420_2026_3204_MOESM1_ESM.pdf]

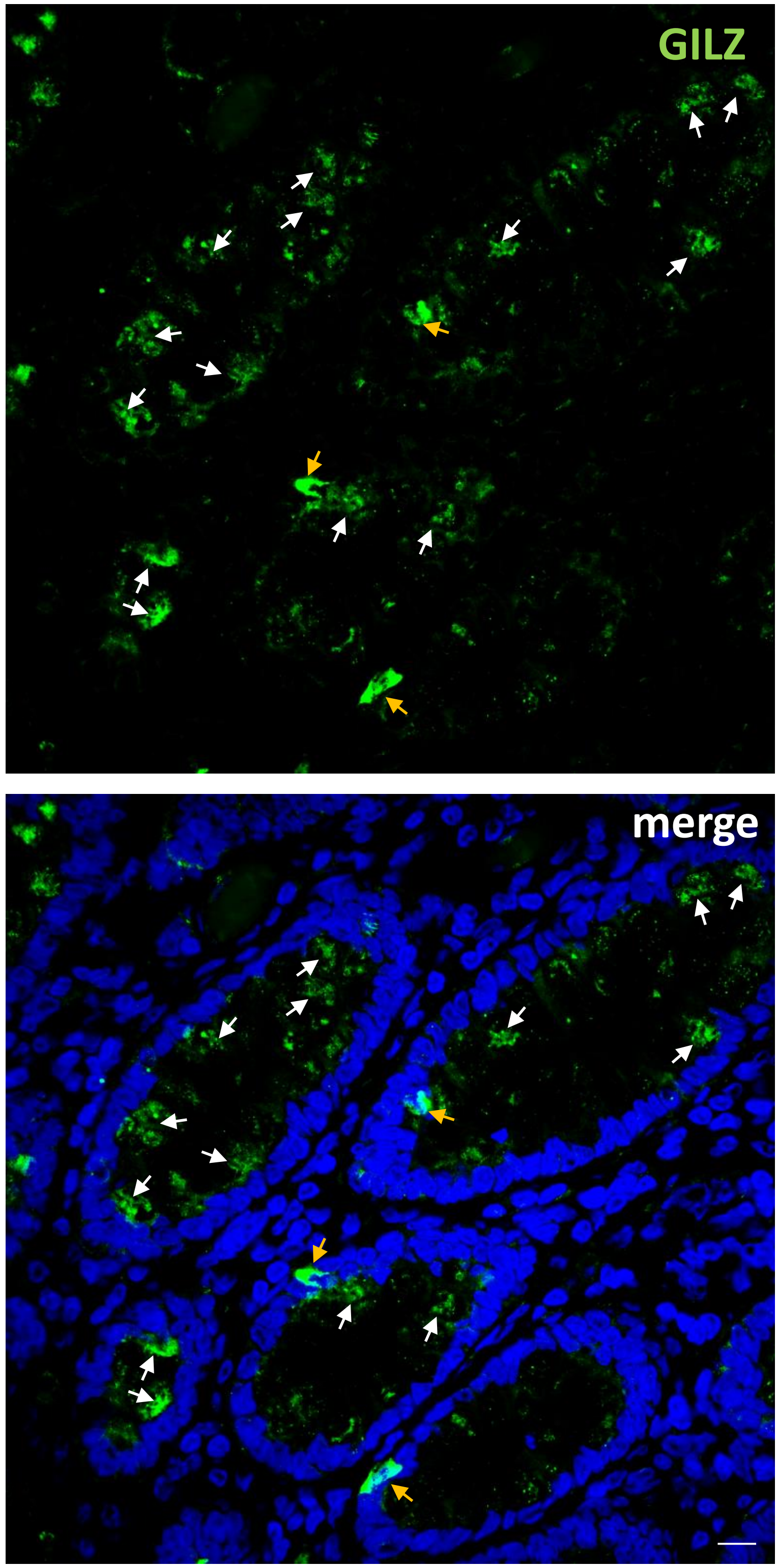

Supplementary Figure 1: confocal imaging of a representative human colon section stained with anti-GILZ antibody. White arrows point at goblet cells, yellow arrows point at cells with apico-basal polarity (scale bar 10µm). Nuclei were counterstained with DAPI.
