# Supplementary material for: Glucocorticoid-induced Leucine Zipper (GILZ) is a novel secreted protein by intestinal L-cells and is dysregulated during active ulcerative colitis
Source: Cell Death Discov. 2026 Jun 17;12:348. doi: 10.1038/s41420-026-03204-w (PMC13518978; doi:10.1038/s41420-026-03204-w)
Supplement: Supplementary file 4 [file 41420_2026_3204_MOESM2_ESM.pdf]

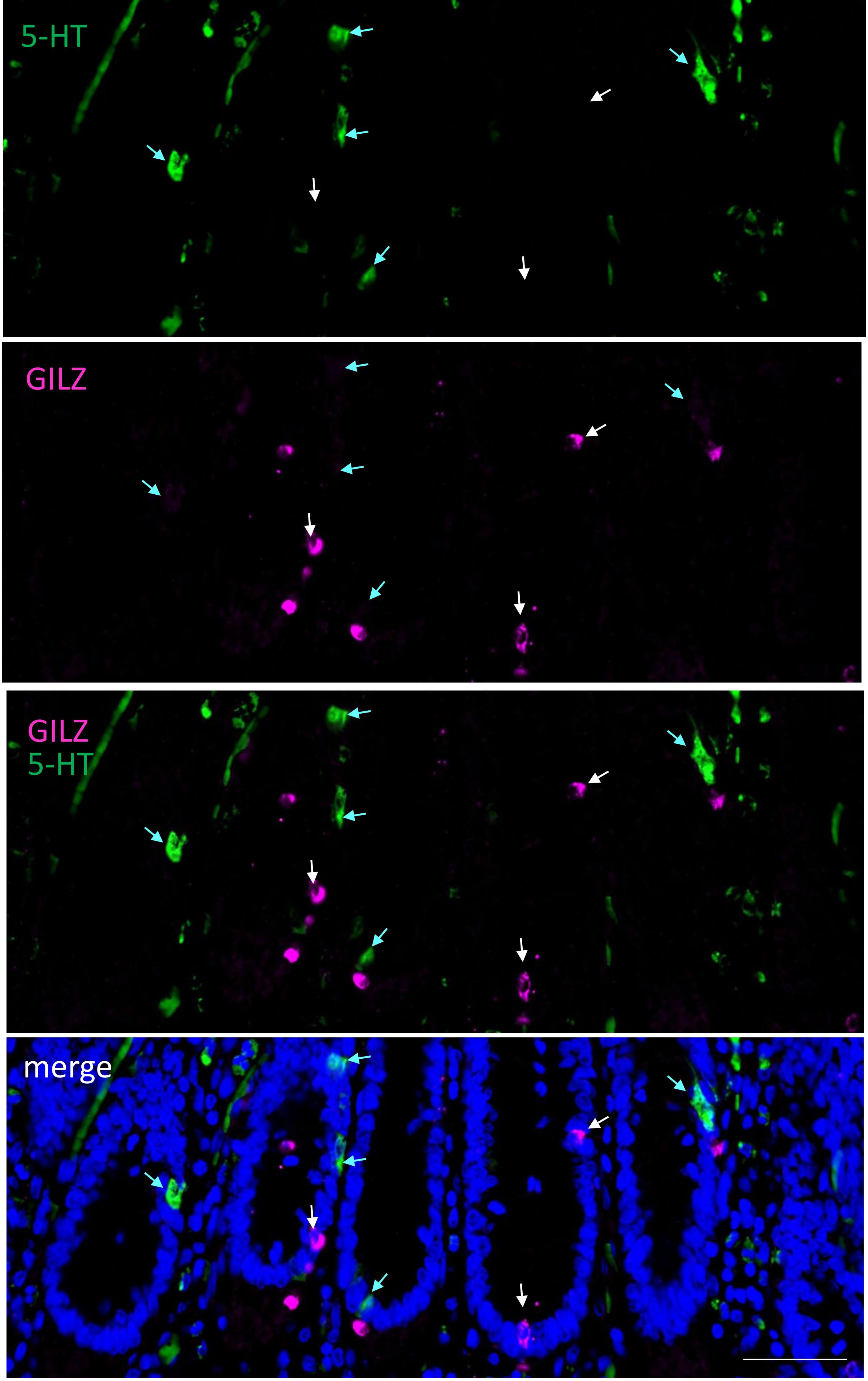
5-HT
GILZ
GILZ
5-HT
merge

Supplementary Figure 2: confocal image of a colon section stained with anti-GILZ (pink) and anti-5HT (green) Abs. Light blue arrows point at $GILZ^{low}$/5-HT^{+} cells, whereas white arrows indicate $GILZ^{high}$/5-HT^{-}. Nuclei were stained with DAPI. Scale bar: 50μm.
