# Supplementary material for: Glucocorticoid-induced Leucine Zipper (GILZ) is a novel secreted protein by intestinal L-cells and is dysregulated during active ulcerative colitis
Source: Cell Death Discov. 2026 Jun 17;12:348. doi: 10.1038/s41420-026-03204-w (PMC13518978; doi:10.1038/s41420-026-03204-w)
Supplement: Supplementary file 5 [file 41420_2026_3204_MOESM3_ESM.pdf]

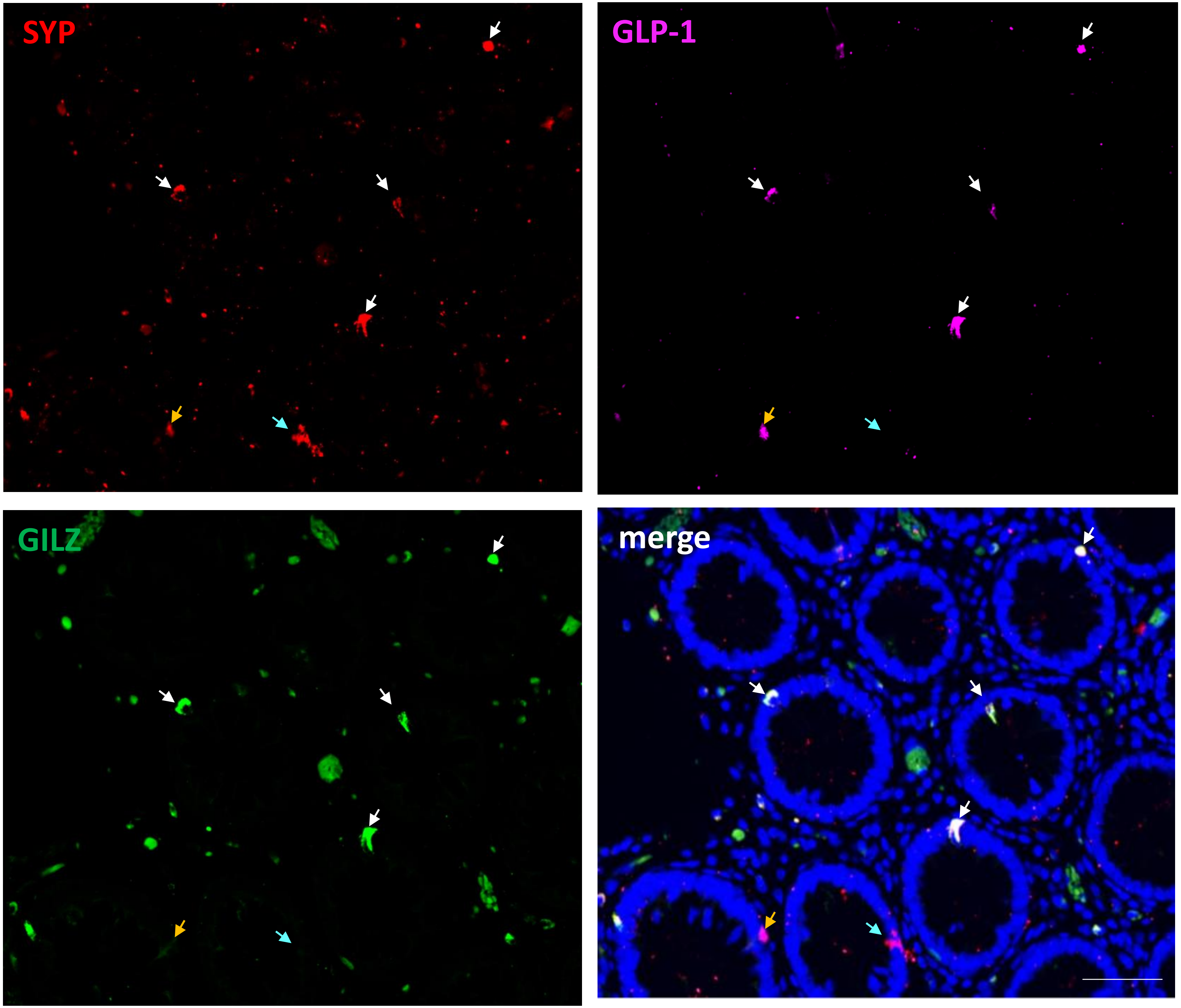
SYP
GLP-1
GILZ
merge

Supplementary Figure 3: representative confocal image of a colon section from a healthy individual. All cells identified by arrows are SYP^{+}. Light blue arrows indicate $GILZ^{-}$/$GLP\text{-}1^{-}$ cells, whereas yellow arrows point at $GILZ^{-}$/$GLP\text{-}1^{+}$ cells. Nulcei were counterstained with DAPI. Scale bar: 50µm.
