# Supplementary material for: Glucocorticoid-induced Leucine Zipper (GILZ) is a novel secreted protein by intestinal L-cells and is dysregulated during active ulcerative colitis
Source: Cell Death Discov. 2026 Jun 17;12:348. doi: 10.1038/s41420-026-03204-w (PMC13518978; doi:10.1038/s41420-026-03204-w)
Supplement: Supplementary file 6 [file 41420_2026_3204_MOESM4_ESM.pdf]

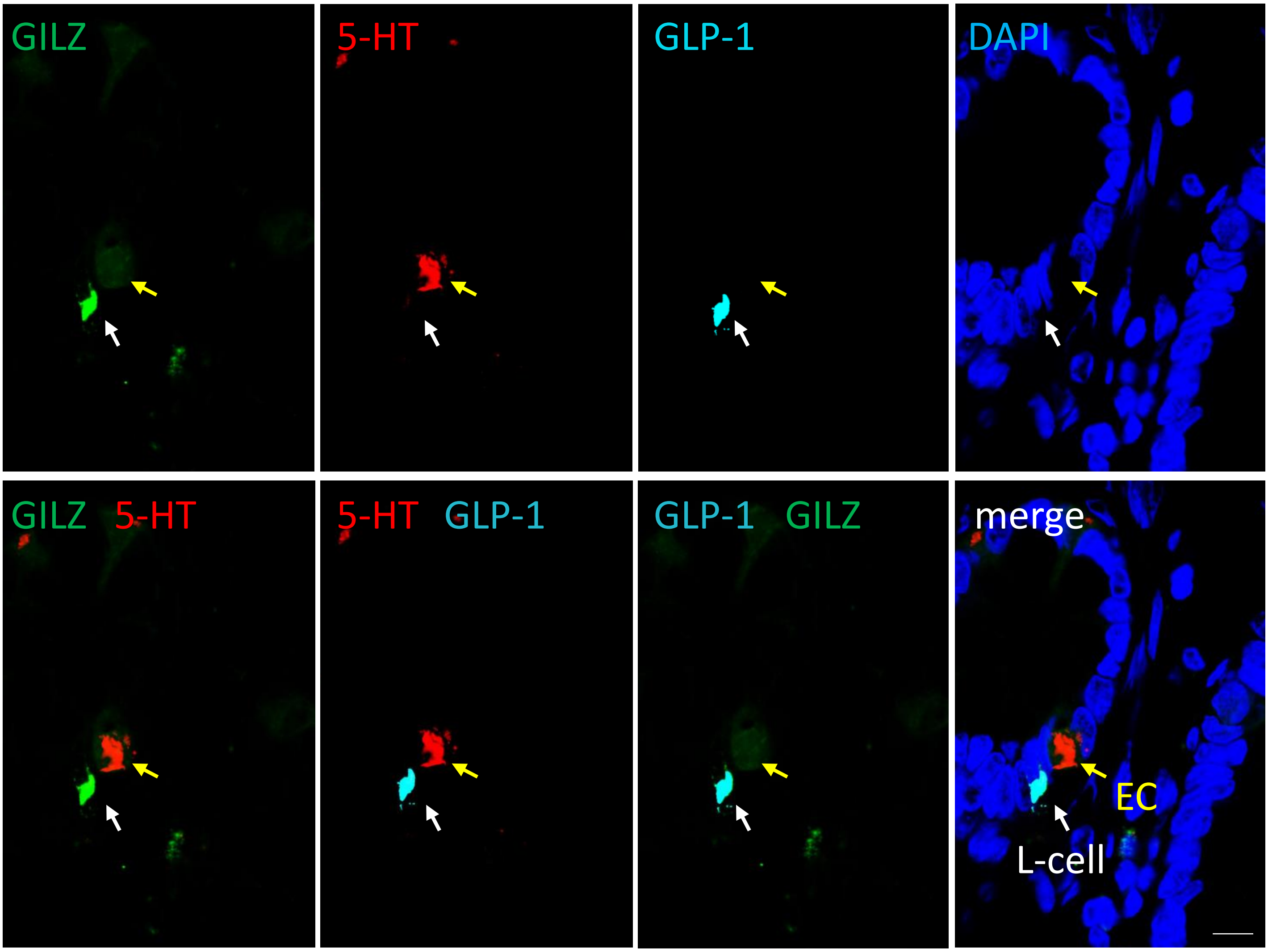

Supplementary Figure 4:Representative image of a human colon section: triple staining with Ab anti-GILZ (green), anti-GLP-1 (light blue), and anti-5-HT (red). The white arrowhead points at a L-cell GLP-1^{+}, whereas the yellow arrowhead points at an enterochromaffin cell (EC) 5-HT^{+}. Nuclei were counterstained with DAPI. Scale bar: 10µm.
