# Supplementary material for: Glucocorticoid-induced Leucine Zipper (GILZ) is a novel secreted protein by intestinal L-cells and is dysregulated during active ulcerative colitis
Source: Cell Death Discov. 2026 Jun 17;12:348. doi: 10.1038/s41420-026-03204-w (PMC13518978; doi:10.1038/s41420-026-03204-w)
Supplement: Supplementary file 7 [file 41420_2026_3204_MOESM5_ESM.pdf]

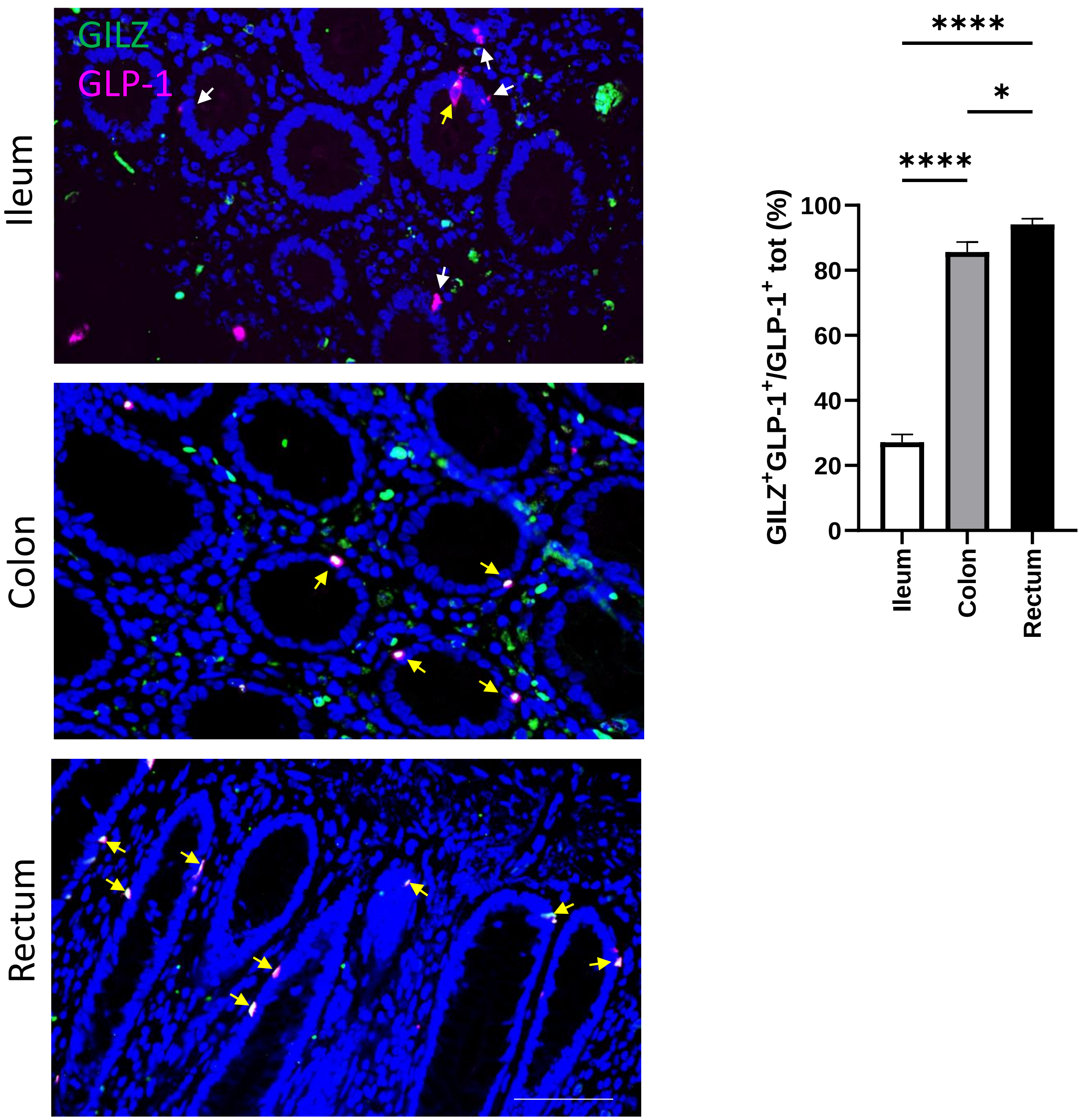

Supplementary Figure 5. Representative confocal images of ileal, colonic and rectal sections from healthy individuals. Sections were double stained with the indicated Abs. Cells were counted in 11, 20, and 12 HPFs in ileum (*n*=5), colon (*n*=7), and rectum (*n*=5) sections, respectively. The percentages of $GILZ^+/GLP\text{-}1^+$ cells are reported in the graph. Scale bar: 100µm. * $p<0.05$, **** $p<0.0001$
