# Supplementary material for: Glucocorticoid-induced Leucine Zipper (GILZ) is a novel secreted protein by intestinal L-cells and is dysregulated during active ulcerative colitis
Source: Cell Death Discov. 2026 Jun 17;12:348. doi: 10.1038/s41420-026-03204-w (PMC13518978; doi:10.1038/s41420-026-03204-w)
Supplement: Supplementary file 8 [file 41420_2026_3204_MOESM6_ESM.pdf]

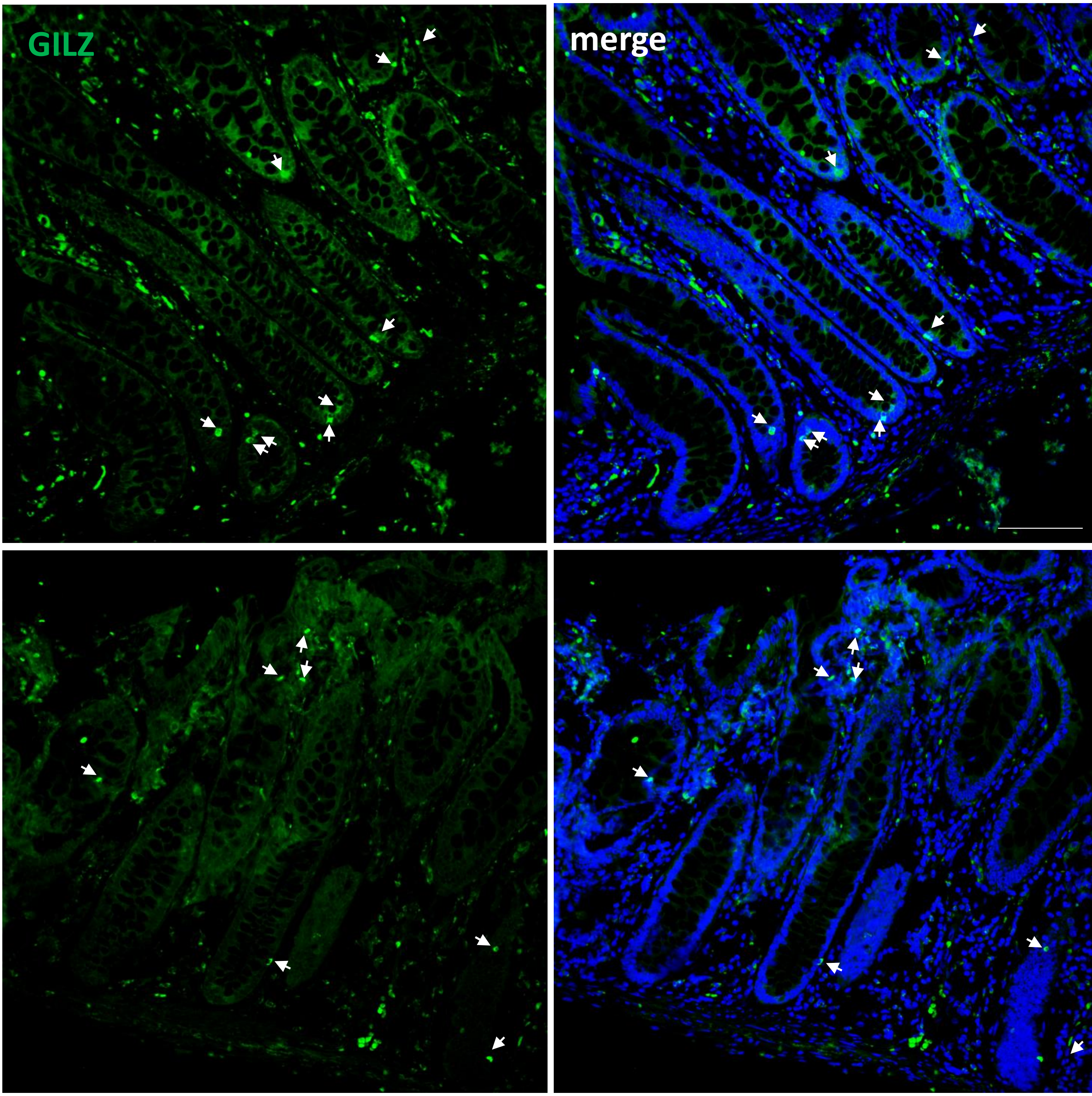

Supplementary Figure 6. Two representative fields (top and bottom panels) of GILZ staining (green). The distribution pattern of GILZ spans from the basal to the apical part of the crypt. Nuclei were counterstained with DAPI. Scale bar: 100 μm.
