# Supplementary material for: Glucocorticoid-induced Leucine Zipper (GILZ) is a novel secreted protein by intestinal L-cells and is dysregulated during active ulcerative colitis
Source: Cell Death Discov. 2026 Jun 17;12:348. doi: 10.1038/s41420-026-03204-w (PMC13518978; doi:10.1038/s41420-026-03204-w)
Supplement: Supplementary file 9 [file 41420_2026_3204_MOESM7_ESM.pdf]

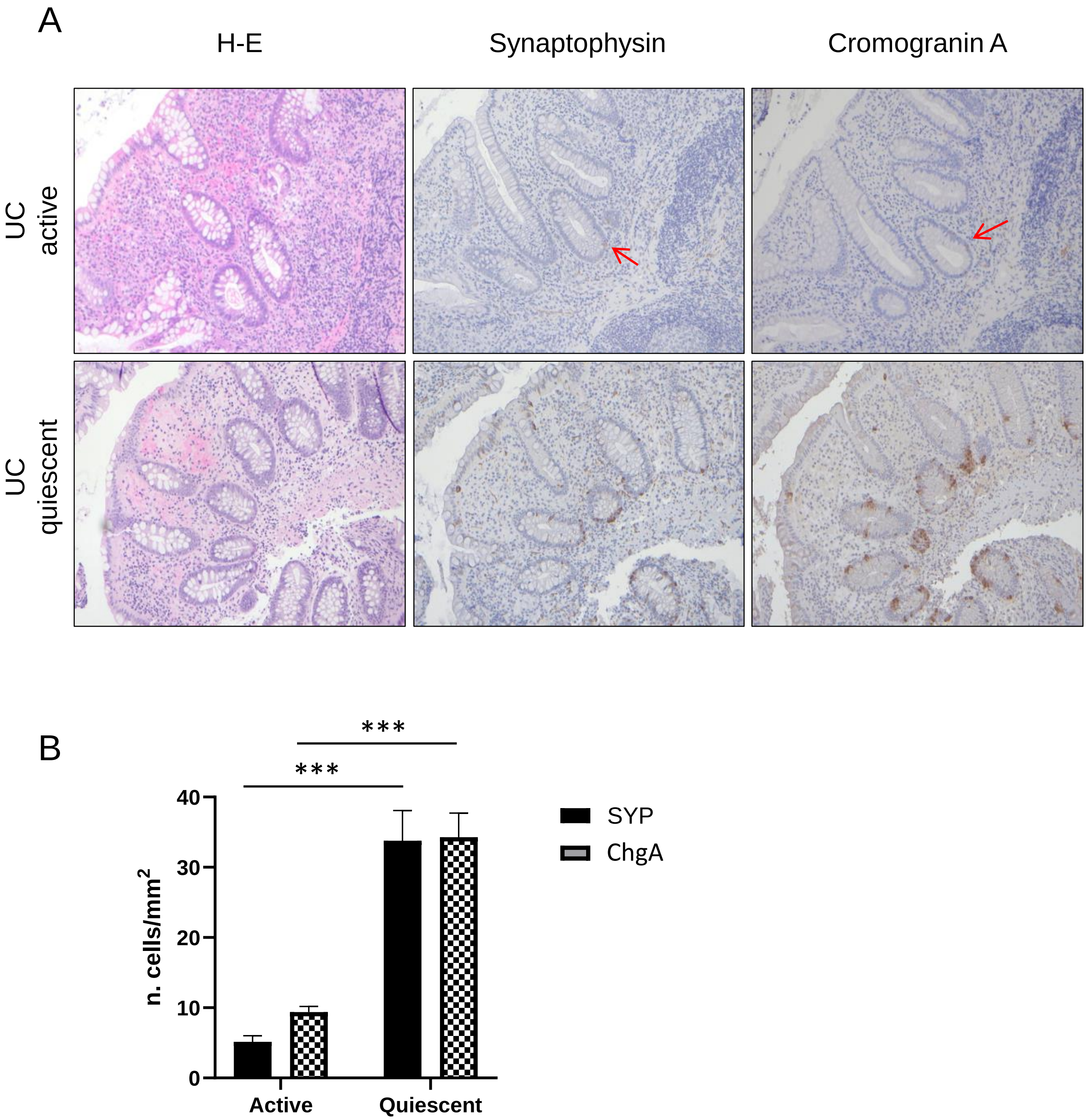

Supplementary Figure 7: immunohistochemistry of SYP and ChgA in active and quiescent UC biopsies. A. Representative images of active and quiescent UC, with neutrophil infiltration and cryptitis in the former. Red arrows point at rare SYP^+ and $ChgA^+$ EEC in active UC. B. Cell counts of SYP^+ and $ChgA^+$ positive EEC. Values represent the mean ± SE. *** $p<0.001$
