# Supplementary material for: Glucocorticoid-induced Leucine Zipper (GILZ) is a novel secreted protein by intestinal L-cells and is dysregulated during active ulcerative colitis
Source: Cell Death Discov. 2026 Jun 17;12:348. doi: 10.1038/s41420-026-03204-w (PMC13518978; doi:10.1038/s41420-026-03204-w)
Supplement: Supplementary file 10 [file 41420_2026_3204_MOESM8_ESM.pdf]

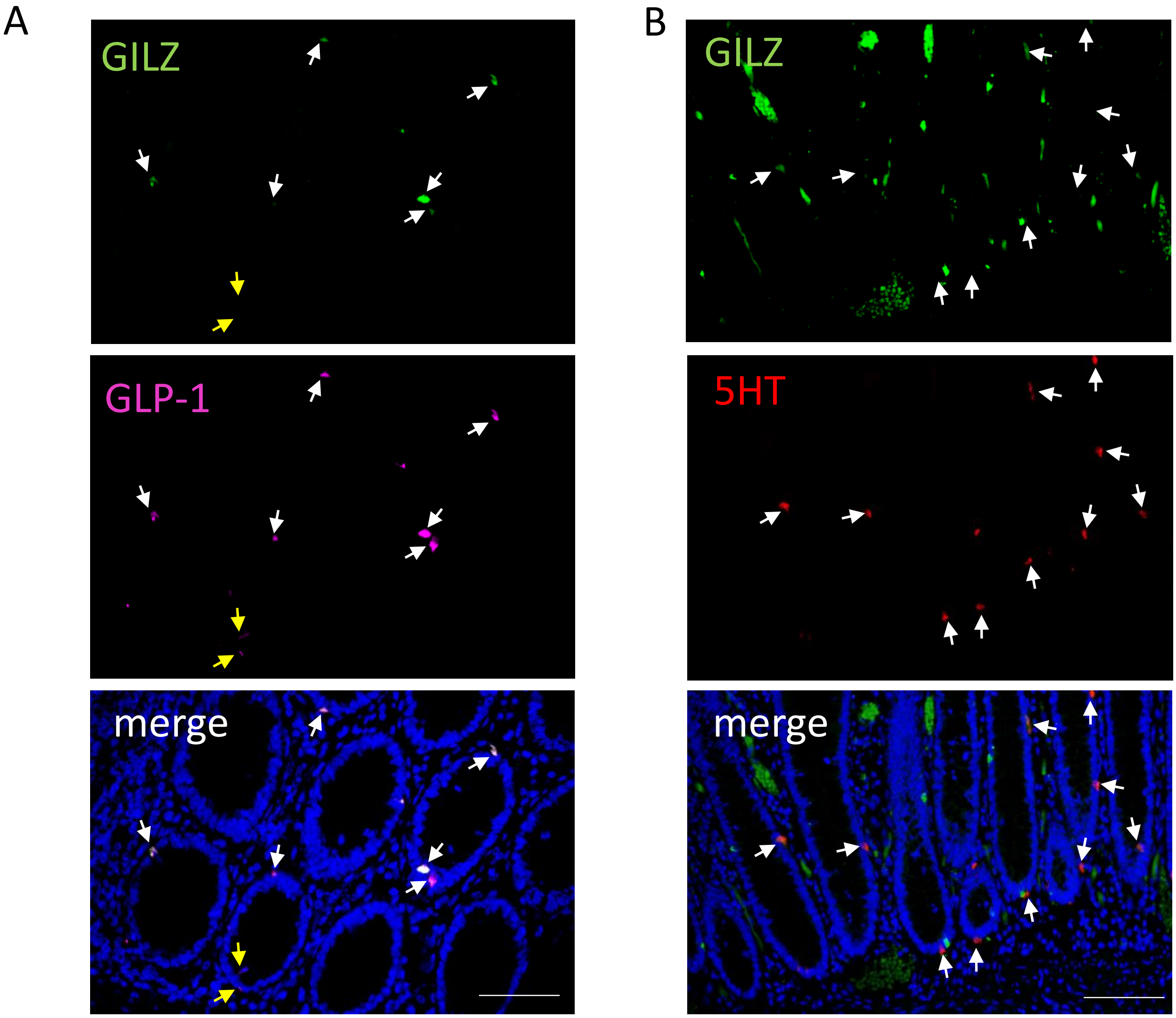

Supplementary Figure 8: confocal images of healthy colon sections. A. Immunofluorescence of a stained colon with anti-GILZ (green) and anti-GLP-1 (pink) Abs. Yellow arrows point at GILZ$^-$/GLP-1$^+$ cells, whereas white arrows indicate GILZ$^+$/GLP-1$^+$ cells. B. Immunofluorescence of a stained colon with anti-GILZ (green) and anti-5HT (red) Abs. Arrows point at 5HT$^+$/GILZ$^+$ cells. Nuclei were stained with DAPI. Scale bars: 100µm.
