# Supplementary material for: Glucocorticoid-induced Leucine Zipper (GILZ) is a novel secreted protein by intestinal L-cells and is dysregulated during active ulcerative colitis
Source: Cell Death Discov. 2026 Jun 17;12:348. doi: 10.1038/s41420-026-03204-w (PMC13518978; doi:10.1038/s41420-026-03204-w)
Supplement: Supplementary file 11 [file 41420_2026_3204_MOESM9_ESM.pdf]

A

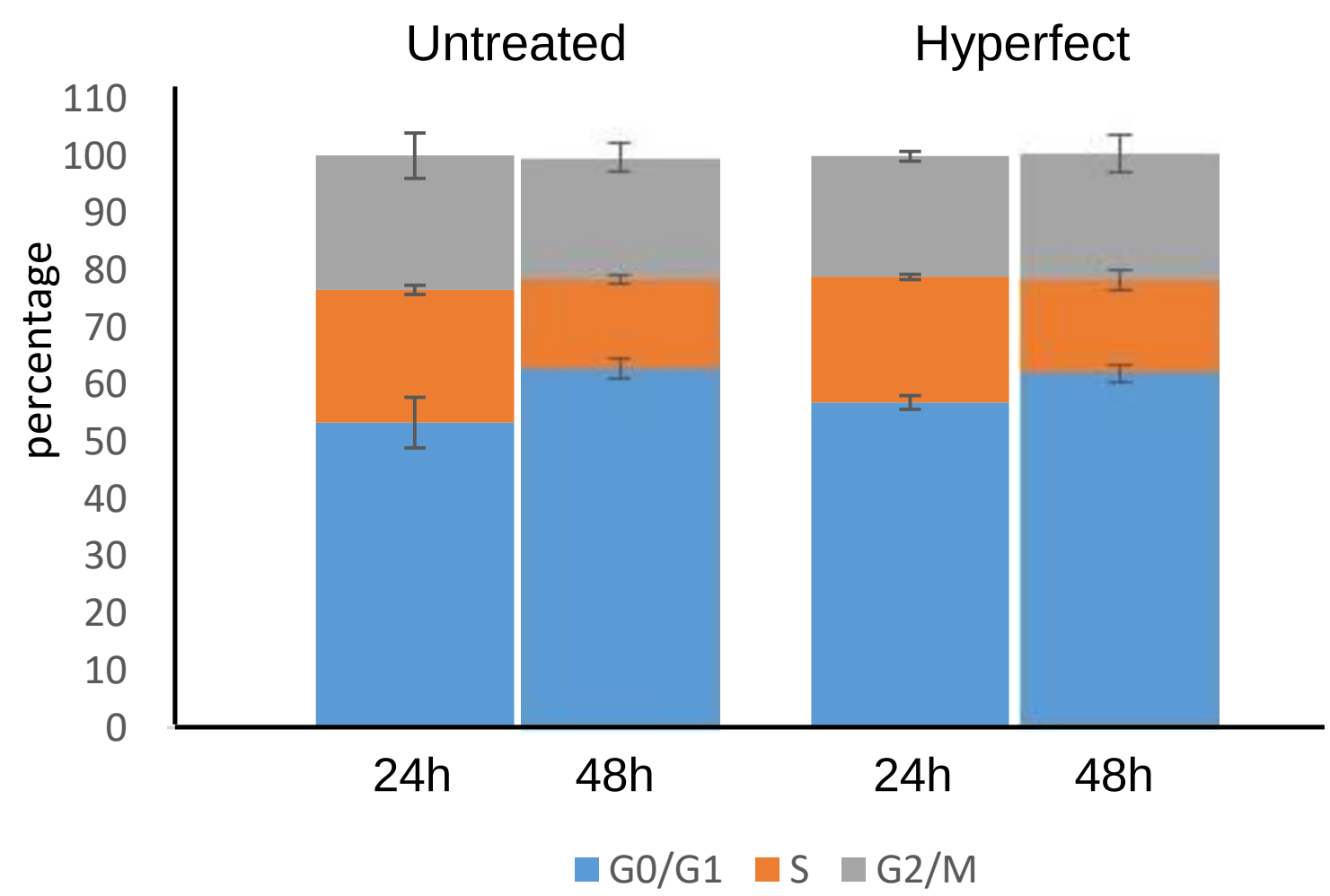

B

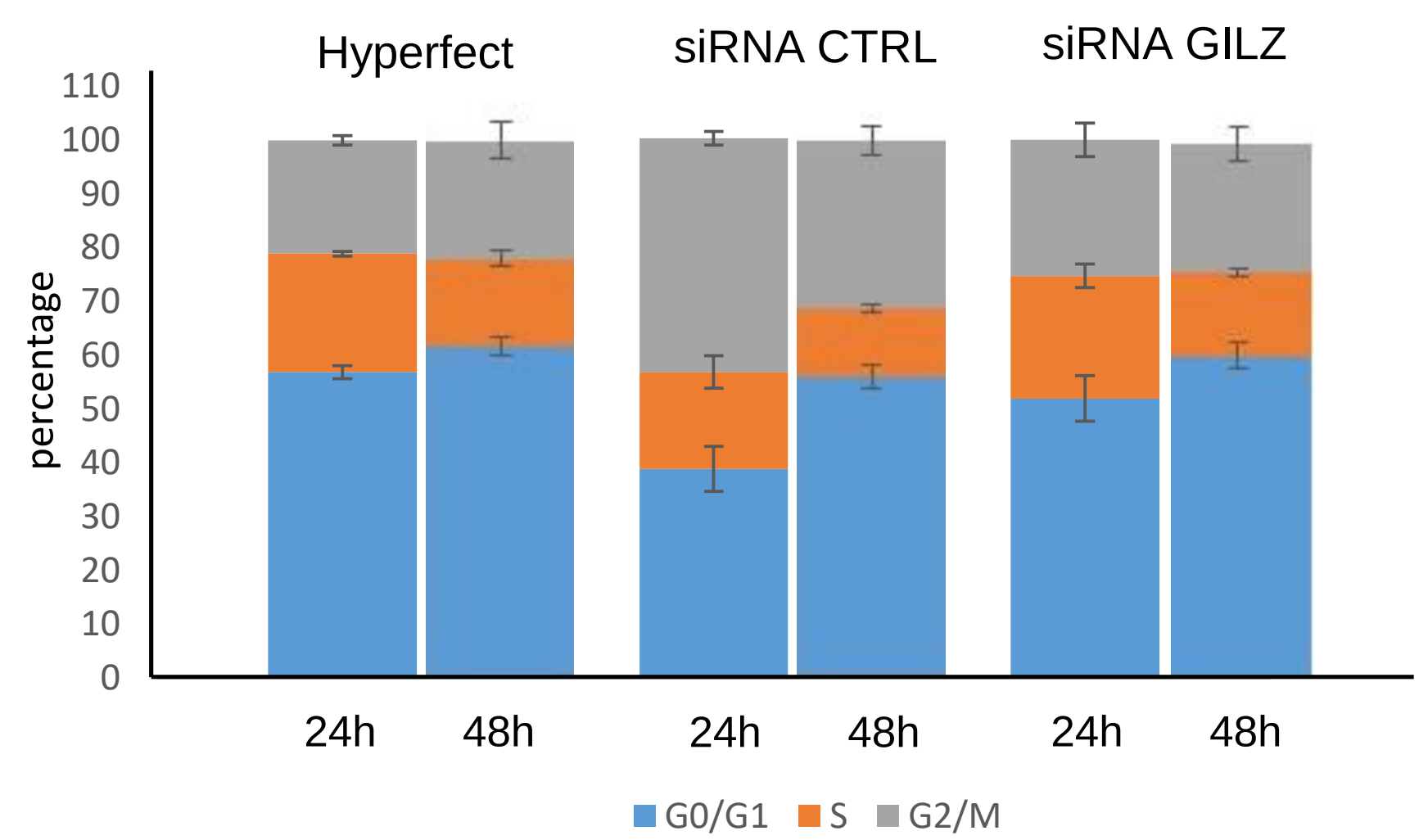

24h

vs	Hyperfect	siRNA-CTRL	siRNA-GILZ
Hyperfect		G0/G1 **<br>S **ns**<br>G2/M ****	**ns**
siRNA-CTRL			G0/G1 **<br>S *<br>G2/M ****

48h

vs	Hyperfect	siRNA-CTRL	siRNA-GILZ
Hyperfect		G0/G1 *<br>S **<br>G2/M **	**ns**
siRNA-CTRL			G0/G1 *<br>S **<br>G2/M *

C

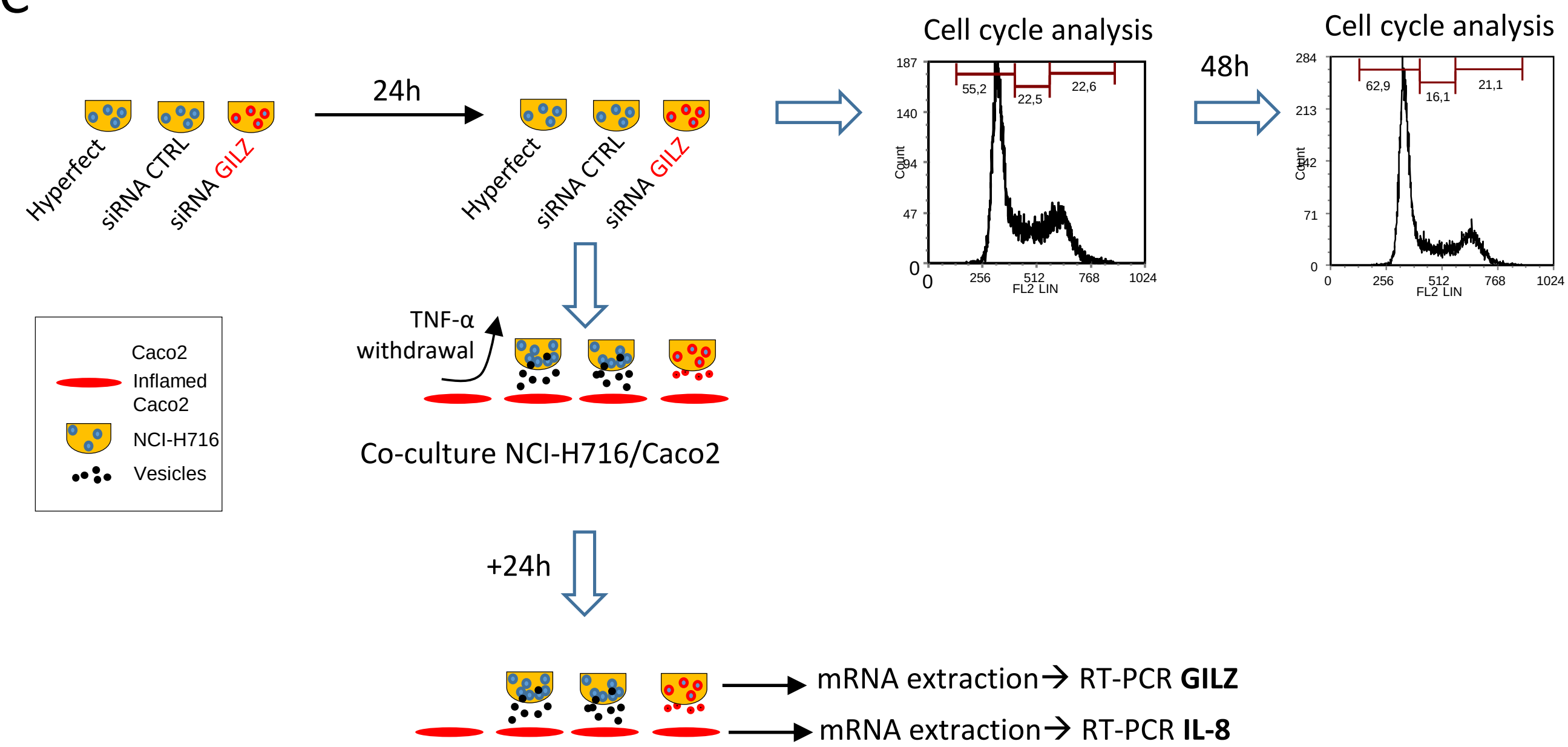

Supplementary Figure 9: Cell cycle phases of NCI-H716 cells in silencing experiments. A. Untreated and Hyperfect-treated NCI-H716 cells were analysed after 24 and 48h from silencing. No differences in G0/G1, S and G2/M phases (percentage of total cells) were observed. B. Hyperfect, siRNA CTRL and siRNA GILZ treated cells were analysed after 24 and 48h from silencing. siRNA CTRL displayed a reduced percentage of cells significantly after 24 and 48h in various cell cycle phases. Significance across the groups is reported in the below table for each single phase. C. Schematic description of the silencing experiments
